# Viral and metabolic controls on high rates of microbial sulfur and carbon cycling in wetland ecosystems

**DOI:** 10.1186/s40168-018-0522-4

**Published:** 2018-08-07

**Authors:** Paula Dalcin Martins, Robert E. Danczak, Simon Roux, Jeroen Frank, Mikayla A. Borton, Richard A. Wolfe, Marie N. Burris, Michael J. Wilkins

**Affiliations:** 1 Department of Microbiology, The Ohio State University, Columbus, OH USA; 2 Department of Energy, Joint Genome Institute, Walnut Creek, CA USA; 3 Department of Microbiology, Radboud University Nijmegen, Nijmegen, The Netherlands; 4 School of Earth Sciences, The Ohio State University, Columbus, OH USA

**Keywords:** Sulfate reduction, Methane, Wetlands, Viruses, Alcohols, C1 metabolism

## Abstract

**Background:**

Microorganisms drive high rates of methanogenesis and carbon mineralization in wetland ecosystems. These signals are especially pronounced in the Prairie Pothole Region of North America, the tenth largest wetland ecosystem in the world. Sulfate reduction rates up to 22 μmol cm^−3^ day^−1^ have been measured in these wetland sediments, as well as methane fluxes up to 160 mg m^−2^ h^−1^—some of the highest emissions ever measured in North American wetlands. While pore waters from PPR wetlands are characterized by high concentrations of sulfur species and dissolved organic carbon, the constraints on microbial activity are poorly understood. Here, we utilized metagenomics to investigate candidate sulfate reducers and methanogens in this ecosystem and identify metabolic and viral controls on microbial activity.

**Results:**

We recovered 162 *dsrA* and 206 *dsrD* sequences from 18 sediment metagenomes and reconstructed 24 candidate sulfate reducer genomes assigned to seven phyla. These genomes encoded the potential for utilizing a wide variety of electron donors, such as methanol and other alcohols, methylamines, and glycine betaine. We also identified 37 *mcrA* sequences spanning five orders and recovered two putative methanogen genomes representing the most abundant taxa—*Methanosaeta* and *Methanoregulaceae*. However, given the abundance of *Methanofollis*-affiliated *mcrA* sequences, the detection of F420-dependent alcohol dehydrogenases, and millimolar concentrations of ethanol and 2-propanol in sediment pore fluids, we hypothesize that these alcohols may drive a significant fraction of methanogenesis in this ecosystem. Finally, extensive viral novelty was detected, with approximately 80% of viral populations being unclassified at any known taxonomic levels and absent from publicly available databases. Many of these viral populations were predicted to target dominant sulfate reducers and methanogens.

**Conclusions:**

Our results indicate that diversity is likely key to extremely high rates of methanogenesis and sulfate reduction observed in these wetlands. The inferred genomic diversity and metabolic versatility could result from dynamic environmental conditions, viral infections, and niche differentiation in the heterogeneous sediment matrix. These processes likely play an important role in modulating carbon and sulfur cycling in this ecosystem.

**Electronic supplementary material:**

The online version of this article (10.1186/s40168-018-0522-4) contains supplementary material, which is available to authorized users.

## Background

Small inland waters are being increasingly recognized as playing an oversized role in greenhouse gas emissions—especially methane (CH_4_) and carbon dioxide (CO_2_). Very small ponds account for 8.6% of the surface areas of lakes and ponds globally yet contribute to 15.1% of CO_2_ emissions and 40.6% of diffusive CH_4_ emissions to the atmosphere [1]. The Prairie Pothole Region (PPR) is the tenth largest wetland ecosystem in the world [2], spanning five US states in the Upper Midwest and three Canadian provinces. This ecosystem contains millions of small depressional wetlands that were formed during the retreat of ice sheets at the end of the Wisconsin glaciation and that now play important ecological roles in waterfowl breeding, retaining surface runoff, nutrient cycling, and pesticide degradation [3, 4]. More recently, pore waters in these wetland sediments have been shown to contain extremely high concentrations of both dissolved organic carbon [5, 6] and diverse sulfur species [7], while some of the highest methane fluxes from wetlands in North America have been measured from this ecosystem [8]. Finally, PPR wetland sediments host some of the highest sulfate reduction rates (SRRs) ever recorded [9], suggesting that this process likely accounts for a large proportion of sediment carbon mineralization.

In such systems, the availability of carbon substrates is likely to play a critical role in controlling the rate of microbial activity. For instance, previous analyses of pore fluids from wetlands in the PPR revealed temporal changes in labile carbon pools (as inferred from fluorescence data), which were associated with primary productivity in the overlying water column occurring in late summer [6]. More recently, we reported the presence of high concentrations of alcohols in pore fluids, while organic acids and methylamines have also been detected [9]. Collectively, variability in carbon compound bioavailability may result in differential microbial activities, as shown recently in a study that identified varying microbial responses to inputs of autochthonous and allochthonous carbon to lake sediments [10]. Moreover, the availability of “non-competitive” substrates (i.e., compounds only available to a particular functional guild of microorganisms) has previously been shown to enable co-occurrence of reductive microbial metabolisms that might otherwise be thermodynamically inhibited [11, 12].

In addition to geochemical constraints, the viral activity may also play a key role in shaping microbial abundances and activities in wetland ecosystems. Viruses affect community turnover and resource availability via a range of interactions with their bacterial hosts. For example, viruses may act as a top-down control on microorganisms, impacting bacterial densities, as well as a bottom-up control through virus-mediated cell lysis and the associated release of labile host contents. Studies in marine aquatic systems have estimated that such cell lysis events drive the release of up to 10^9^ tons of carbon every day [13]. More generally, viral predation is thought to be an important control on community structure, especially for fast-growing dominant microbial strains [14, 15]. Given the high sulfate reduction rates previously measured in PPR sediments, we anticipate that viral predation may represent an important process controlling rates of carbon mineralization in this ecosystem.

Despite the abundance of geochemical data for wetland sediments in the PPR, and the importance of these ecosystems in regional carbon and sulfur cycling, the underlying microbial populations driving these processes and the potential controls on their activity are poorly understood. Here, we provide the first characterization of such populations and controls using genome-resolved metagenomics. From 18 metagenomes, we recovered key gene sequences and microbial draft genomes from organisms likely responsible for sulfate reduction and methane production. Moreover, we predicted that viral populations target candidate sulfur- and carbon-cycling microbial hosts and investigated spatiotemporal dynamics in viral and host abundances and community structure. The ability of phylogenetically and functionally diverse groups of sulfate reducers and methanogens to use a wide range of substrates may at least partly explain the high levels of biogeochemical activity measured in PPR wetland sediments. Additional linkages between dominant microorganisms and viruses may represent one control on sulfate reduction and methanogenesis at the ecosystem level.

## Methods

### Sample collection and DNA extractions

Sediment core samples were collected from two adjacent wetlands, P7 and P8, at the United States Geological Survey-managed Cottonwood Lake Study Area near Jamestown, ND, USA [9]. From 16S rRNA gene analyses, 18 representative sediment samples were selected for metagenomic sequencing based on wetland (P7 and P8), season (winter, spring, summer), and depth (1–3, 10–12, and 19–21 cm) (Additional file 1: Table S1). After being stored at − 80 °C, sediments were thawed, and DNA was extracted using the MoBio PowerLyzer Powersoil® DNA Isolation Kit (Mo Bio Laboratories, Inc., Carlsbad, CA, USA) according to the manufacturer’s instructions. Following extraction, nucleic acids were quantified (Additional file 1: Table S1) using a Qubit® Fluorometer (Invitrogen, Carlsbad, CA, USA) and diluted, so ~ 200 ng of DNA per sample was sent for metagenomic sequencing at the DOE Joint Genome Institute. These samples had been previously analyzed using 16S rRNA gene sequencing and pore water measurements of sulfate, sulfide, ferrous iron, methane, methanol, trimethylamine, ethanol, 2-propanol, acetate, acetone, and formate [9]. Here, these geochemical measurements were used as input values for principal component analysis in R [16] in order to illustrate the geochemical differences between P7 and P8.

### DNA sequencing, quality control, and assembly

Genomic DNA libraries with an insert size of 270 bp were sequenced on the Illumina HiSeq 2500 platform, generating paired-end reads (2 × 151 bp). Reads were processed with BBDuk [17] to remove Illumina adapters and primers. Reads containing traces of spike-ins were discarded entirely. Bases with a Phred quality score (*Q*) below 12 were trimmed from both the 5′ and 3′ end of the sequences. Reads smaller than 51 bp or containing more than one ambiguous base (N) were removed (ktrim = r, minlen = 40, minlenfraction = 0.6, mink = 11, tbo, tpe, k = 23, hdist = 1, hdist2 = 1, ftm = 5, maq = 8, maxns = 1, k = 27, trimq = 12, qtrim = rl). The remaining reads were mapped against a masked version of the human reference genome (HG19) using BBMap 35.82 [17] to remove sequences of putative human origin. Reads aligning with more than 93% identity to HG19 were discarded (fast, local, minratio = 0.84, maxindel = 6, tipsearch = 4, bw = 18, bwr = 0.18, usemodulo, printunmappedcount, idtag, minhits = 1). Metagenome assembly was performed using MEGAHIT v1.0.3 [18] using a range of *k*-mers (“--k-list 23,43,63,83,103,123”) at default settings.

### Contig merging and binning

In order to improve assembly and reduce redundancy for binning using differential coverage, the 18 assemblies were merged with Newbler and dereplicated with a custom script, which is part of the MeGAMerge pipeline [19] with default parameters. Only contigs larger than 1500 bp were retained. Reads were mapped back to the final contig set using Bowtie2 [20], from ~ 2.16 billion trimmed, quality-controlled metagenome reads, 33% mapped to the final set of contigs (Additional file 2: Table S2). The generated sequence mapping files were handled and converted as needed using SAMtools 1.6 [21]. Metagenome binning was performed employing three different binning algorithms with default parameters: CONCOCT 0.4.1 [22], MaxBin2 v. 2.2.3 [23], and MetaBAT2 v. 2.10.2 [24]. The three resulting bin sets were supplied to DAS Tool 1.0 [25] for consensus binning and dereplication, generating an optimized set of bins named after their seed bin method. Selected bins from the MetaBAT run before the DAS Tool step were added to the final pool of bins, being named bin.1, bin.2, etc., because some viable bins were lost or lose marker genes during this process despite the overall improvement. Bins were verified manually to ensure the selected bins did not overlap with the post-DAS Tool bins. A single-copy marker gene analysis was performed using CheckM 1.0.7 [26] to assess the quality (completeness and contamination) of the genome bins.

### Identification of viral contigs and construction of a viral OTU table

Viral sequences within our metagenomic dataset likely originate from populations of double-stranded or single-stranded DNA phages, including both lytic phages (intracellular and extracellular) and temperate phages integrated into the microbial chromosome or existing as extrachromosomal elements. VirSorter [27] was used to identify viral contigs in the merged contig set with default parameters: “*Virome db*” as the database, no additional viral sequence to be used as a reference, and no virome decontamination, outputting 29,317 putative viral sequences. Only the highest confidence contig categories 1, 2, 4, and 5 (no. 3 or 6) were included in this study, with categories 4 and 5 being manually curated, resulting in 19,127 sequences. Out of those, 4262 sequences larger than 5000 bp were pooled together and clustered at 95% average nucleotide identity (ANI) over 80% of the contig length [28], resulting in 3344 unique viral seeds. Binning of viral contigs with MetaBAT [24] was unsuccessful, so each viral seed was considered a viral population or viral operational taxonomic unit (vOTU).

Bowtie2 [20] was used to map reads back to viral populations. Reads Per Kilobase per Million mapped reads (RPKM) values for each contig were calculated as the number of mapped reads times 10^9^ divided by the total number of reads times the contig length. A contig was considered to be present in a sample only if at least 75% of the contig length was covered by reads in that sample. The generated vOTU table with viral abundances (RPKM values) in each sample retained 3329 viral contigs and was used as an input for analyses in R using the *vegan* package v.2.4-4 [29]: non-metric multidimensional scaling (NMDS) with *metaMDS*, PERMANOVA (*adonis* function), and *procrustes*/*protest* [30] to correlate a 16S-based microbial NMDS to a metagenomics-based viral NMDS. The 16S rRNA gene-based microbial data has already been published [9], and a subset of these data (18 samples) for which we performed metagenomic sequencing was selected and reanalyzed. The total viral abundance in each sample was calculated as the sum of RPKM values for individual contigs in that sample, and it was used to construct bar charts in R. All figures in this article were edited in Adobe Illustrator version 16.0.0 (Adobe Systems Inc., San Jose, USA).

### Annotation, marker gene analyses, and virally encoded metabolic genes

Marker genes such as *dsrA*, *dsrD*, and *mcrA* were screened using the hidden Markov models (HMMs) from Anantharaman et al. [31] with hmmsearch (HMMER v3.1b2) using the flag “--cut_tc” [32]. The minimum sequence length for DsrA, DsrD, and McrA sequences to be included in gene analyses was 302, 57, and 150 amino acids, respectively. A tree with reference sequences (as described below) was built to select only for reductive-type *dsrA* sequences. To search for *Methanofollis* alcohol dehydrogenases and ribosomal proteins in our dataset, we have used these proteins in the reference genomes NZ_CM001555.1 and NZ_BCNW00000000.1 for BLAST analyses. MttB homolog sequences were recovered from contigs based on protein annotations.

The abundance of these marker genes in each sample was computed as the RPKM value for each marker gene-containing contig, which was calculated as for vOTU abundance. RPKM values were used to build heat maps in R with the function *heatmap.2*, and heat map hierarchical clustering statistical significance was tested using the *pvclust* R package (method.dist = “euclidean”, method.hclust = “complete”, nboot = 10,000). Only approximately unbiased *p* values larger than 95% were considered as significant. The natural logarithm Shannon diversity was calculated in R using the *diversity* function with the *vegan* package [29]. Paired *t* tests were performed in R to test differences in Shannon diversity across the two wetlands.

RPKM values were also used in R (*vegan* package) to test gene/contig abundance differences across samples with PERMANOVA (*adonis* function) and to construct redundancy analyses (RDA) plots. For the latter, abundances were Hellinger transformed with the *decostand* function, and then forward selection of the best environmental variables was applied using *ordistep*, which was performed only if global tests with all variables were significant. Adjusted *R*^2^ and *p* values were reported for significant statistical analyses.

Bins containing marker genes of interest and all viral contigs were gene-called and annotated using an in-house annotation pipeline as previously described [33, 34]. Briefly, genes were called with Prodigal [35] and annotated based on forward and reverse blast hits (minimum 300 bit score threshold for reciprocal matches and 60 for one-way matches) to amino acid sequences in the databases UniRef90 and KEGG, while motifs were analyzed using InterProScan. The taxonomical affiliation of marker genes was inferred from the best BLASTP hit excluding uncultured/environmental sequences. The taxonomical classification of bins was determined based on lineage-specific phylogenetic markers from CheckM [26]. Annotations were used to search for virally encoded metabolic genes in viral contigs based on the following criteria: (i) gene is in the middle of the contig (not the first or last two genes), (ii) contig is clearly viral (contains hallmark phage genes such as tail or capsid protein), (iii) gene occurs at least in three viral contigs, and (iv) gene product can only act in the host cell metabolism and could not be used in the viral cycle (DNA replication, capsid formation, etc). No genes met these criteria.

### Construction of phylogenetic trees

For phylogenetic trees, amino acid sequences were aligned with MUSCLE v 3.8.31 [36], and columns with at least 95% gaps were removed with Geneious® 9.0.5 [37]. Trees were built as previously described [38] using Protpipeliner, an in-house pipeline that curates alignments with GBLOCKS [39], selects the best model with ProtTest v. 3.4 [40], and provides a tree using RAxML v. 8.3.1 with a 100 bootstraps [41]. The *mcrA*, *dsrA*, and *mttB* trees were built under the LG + I + G model of evolution, while the *dsrD* tree, under the WAG + G model. All trees were visualized with iToL [42].

### Taxonomic classification of viruses

Viral taxonomy was assigned using vConTACT [43]. Briefly, viral proteins were obtained from Prodigal as part of the aforementioned annotation pipeline and combined with the viral protein database “PC_aminoacid_database_REFS.faa” from CyVerse [44]. Headers were modified to avoid underscores and contain up to 30 characters and were used to construct the “protein.csv” file in windows .csv format. An all-versus-all BLAST was run with the following parameters: “outfmt 6 -evalue 1e-3 -max_target_seqs 239262.” The maximum number of target sequences was set as the total number of headers in the amino acid fasta file to avoid losing information given that, by default, BLAST outputs only the best 500 hits. From this point, data was uploaded into CyVerse, and both apps *vcontact_pcs 0.1.60* and *vcontact 0.1.60* were run with default parameters (link significitivity, 1; significativity threshold, 1; module inflation, 5; module significativity, 1; link proportion, 0.5; inflation, 2; module shared min, 3). The output file “cc_sig1.0_mcl2.0.ntw” was downloaded and imported into Cytoscape 3.1.1 [45], while the attribute file was manually constructed and imported into Cytoscape as well. The prefuse force-directed layout was used and the app *clusterMaker* was run with the “MCL cluster” option and the following parameters: granularity 2.0, array sources “c,” edge weight conversion “none,” edge cutoff 1.001, assume edges are undirected, assume loops before clustering, weal edge weight pruning threshold 1E−15, number of interactions 16, maximum residual value 0.001, create groups (metanodes) with results, and create new clustered network. Modules containing only reference viral genomes were removed, and viral classification was retrieved from the module table. The classification of five contigs that clustered with virophage reference sequences was manually curated. We could not identify any virophage marker gene on these contigs, suggesting that this affiliation stemmed from genes not specific to virophages but potentially shared across multiple viral groups. Therefore, we conservatively opted to consider these sequences as “unclassified” in our subsequent analyses.

### Viral identification in other datasets

We attempted to identify viral contigs similar to the novel viral sequences in this study from two database collections: the Global Ocean Virome (GOV) [46], which contains sequences from the Tara Oceans Expeditions and Malaspina, and the VirSorter curated dataset [47], which contains sequences from RefSeq (January 2015), Whole Genome Shotgun, Microbial Dark Matter, and SUP05 databases. For a viral contig to be identified via BLAST in other databases, we required a minimum of 70% identity over 90% of the contig length, a minimum bit score of 50, and a maximum *e* value of 0.001, according to the previously published thresholds [46].

### Linking viruses to hosts

Four methods were used to infer putative virus-host links: BLAST [48], to identify prophages in microbial bins; CRASS 1.0.1 [49], to look for CRISPR array sequences (direct repeats and spacers), which are then compared to viral contigs; VirHostMatcher 1.0 [50] and WIsH 1.0 [51], to infer links based on *k*-mer frequencies in viral and host genomes. Viral contigs were blasted against microbial bins with the following thresholds for host prediction: minimum 75% of viral contig length, 70% similarity, 50 minimum bit score, and 0.001 maximum *e* value. CRASS was run on quality-controlled, trimmed metagenome reads with “-n 5000” and “-e 1e-8” as options. The output crass_summary_DR1.txt and crass_summary_SP1.txt files were used to manually verify which direct repeats in microbial genomes matched spacers corresponding to viral contigs. Direct repeats and spacers were aligned to microbial and viral contigs, respectively, in Geneious® 9.0.5 [37], where only one mismatch was allowed and an alignment over the full spacer was required for host prediction. VirHostMatcher was run with default parameters, and d2* values ≤ 0.2 were considered a link. WIsH was run with default parameters against our microbial genome dataset and microbial genomes from the IMG database [52]. Links were inferred when *p* < 0.001, then the lowest common ancestor of the best five hits was taken as the host.

## Results

### PPR wetlands host diverse populations of sulfate-reducing microorganisms

**Fig. 1:**
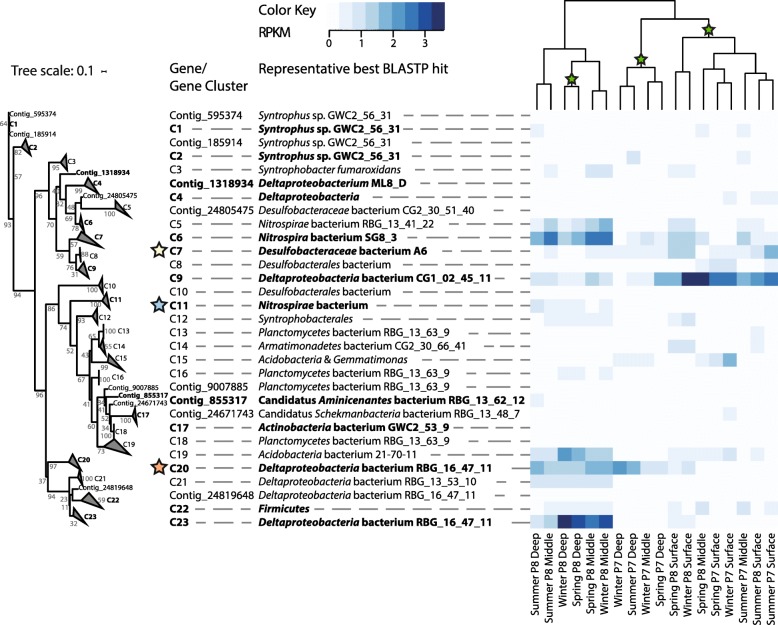
*dsrD* phylogenetic affiliation and abundance per sample. The RAxML tree was constructed using 206 amino acid sequences. The gene or gene cluster (C1–23) affiliation was inferred from the (representative) best BLASTP hit. Bolded names represent *dsrD* present in reconstructed genomes. The yellow, blue, and orange stars indicate *dsrD* in genomes represented in Fig. 2. For the heat map, *dsrD*-containing contig RPKM values were used as input. Clusters are represented by the sum of RPKM values. The statistical significance of hierarchical clustering branches is indicated by green stars (pvclust, approximately unbiased *p* < 0.05). Additional file 4: Figure S1 is an expanded version of this figure, displaying each one of the 206 sequences

Previously, we reported extremely high sulfate reduction rates in sediments collected from PPR wetlands [9]. In order to identify sulfate-reducing microorganisms that could account for these rates, metagenomic data was searched for two marker genes: the traditional reductive-type *dsrA* gene and *dsrD*. Despite not being a functional maker gene and having an unknown function, *dsrD* is generally absent from sulfur oxidizers that utilize the oxidative-type *dsrA* pathway [53] and has previously been used in metagenomic sulfate reduction studies [54]. A notable exception is *Desulfurivibrio alkaliphilus*, which oxidizes sulfur and encodes *dsrD* [55]. Therefore, we have used *dsrD* to tentatively assign a sulfur metabolism in conjunction with analyses of other *dsr* genes. In total, we recovered 162 reductive-type *dsrA* sequences (Additional file 3: Table S3) and 206 *dsrD* sequences, with the taxonomy (per best BLASTP hit of DsrD) of the sequences spanning ten bacterial phyla (Fig. 1). RPKM values of *dsrD*-containing contigs revealed that gene abundances differed significantly between the two wetlands (Additional file 4: Figure S1; PERMANOVA, *F* = 10.627, *p* < 0.001), and redundancy analyses confirmed that wetland was a primary factor constraining the composition and abundance of sulfate-reducing populations (Additional file 5: Figure S2). The same trends were observed for *dsrA*; gene abundances also differed between the two wetlands (Additional file 6: Figure S3; PERMANOVA, *F* = 11.294, *p* < 0.001).

The majority of DsrD amino acid sequences were affiliated with microorganisms within the *Deltaproteobacteria* (127), with smaller numbers of sequences affiliated with *Nitrospirae* (33), *Acidobacteria* (18), *Planctomycetes* (9), *Firmicutes* (8), the candidate phyla *Armatimonadetes* (4), *Gemmatimonadetes* (3), *Aminicenantes* (1) and *Schekmanbacteria* (1), and *Actinobacteria* (2). However, across all samples, the most abundant *dsrD* sequences (inferred from RPKM values) were associated with *Nitrospira* strains (Additional file 4: Figure S1 and Fig. 1). The summing of *dsrD* RPKM values across samples revealed that candidate sulfate-reducing bacteria (SRB) were generally more abundant in wetland P8 than in P7 (Additional file 7: Table S4). Across all samples, the *dsrD*-based Shannon diversity index varied between 2.85 and 4.81, with no statistical difference between the two wetlands (Additional file 7: Table S4).

### Abundant candidate sulfate reducers are metabolically versatile

From metagenomic data, we reconstructed 24 putative SRB metagenome-assembled genomes (MAGs) that contained *dsrD* and/or reductive-type *dsrA* sequences (bolded names in Fig. 1 and Additional file 4: Figure S1; Additional file 8: Table S5 for MAG contamination and completeness). None of these MAGs encoded the sulfur oxidation genes *dsrL*, *soxA*, *soxB*, *soxC*, *soxD*, *soxY*, *soxZ*, *soxX*, or a sulfide quinone oxidoreductase. These MAGs were distributed throughout the *Deltaproteobacteria* (14), *Chloroflexi* (4), *Acidobacteria* (2), *Planctomycetes* (1), *Spirochaetales* (1), candidatus *Aminicenantes* (1), and *Nitrospirae* (1). Versatile metabolic traits were encoded across these genomes. The *Planctomycetes* genome, although very incomplete (~ 24% with 3.5% contamination), encoded genes for the reduction of sulfate (*dsrAB*, *dsrTMKJOP*), nitrate (*narGHI*), nitrite (*nirBD*), and oxygen (subunits of NADH dehydrogenase, succinate dehydrogenase, *aa*_*3*_-type and *cbb*_*3*_-type cytochrome c oxidases, and a complete cytochrome *bd*_*1*_ complex). This genome also exhibited versatility with regard to potential electron donors, encoding a methanol dehydrogenase, glycine betaine utilization *mtg* genes, alcohol dehydrogenases, lactate dehydrogenases, formate dehydrogenase, a variety of genes involved in pyruvate metabolism, and nickel-iron hydrogenases.

**Fig. 2:**
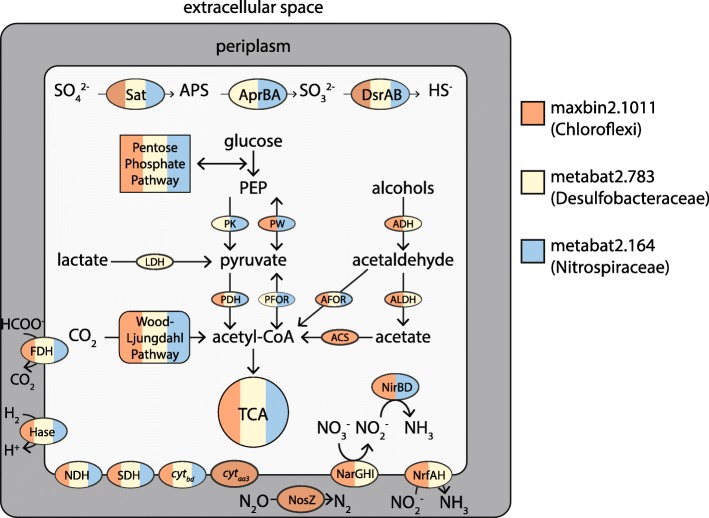
Genome cartoon of three representative candidate sulfate reducers. The cartoon displays metabolic pathways encoded by a *Chloroflexi* (orange), *Desulfobacteraceae* (yellow), and *Nitrospiraceae* (blue) genome. The abbreviations and chemical formulae are as follows: SO_4_^2−^, sulfate; Sat, sulfate adenylyltransferase; APS, adenosine 5′-phosphosulfate; AprBA, APS reductase subunits A and B; SO_3_^2−^, sulfite; DsrAB, dissimilatory sulfite reductase subunits A and B; PEP, phosphoenolpyruvate; PK, pyruvate orthophosphate dikinase, PW: pyruvate water dikinase; ADH, alcohol dehydrogenase; LDH, lactate dehydrogenase; PDH, pyruvate dehydrogenase; PFOR, pyruvate ferredoxin oxidoreductase; AFOR, acetaldehyde ferredoxin oxidoreductase; ALDH, aldehyde dehydrogenase; ACS, acetate synthetase; HCOO^−^, formate; FDH, formate dehydrogenase; CO_2_, carbon dioxide; H_2_, hydrogen; Hase, nickel-iron hydrogenase; H^+^, proton; NDH, NADH dehydrogenase; SDH, succinate dehydrogenase; *cyt*_*bd*_, cytochrome *bd*; *cyt*_*aa3*_, *aa*_*3*_-type cytochrome; TCA, tricarboxylic acid cycle; N_2_O, nitrous oxide; NosZ, nitrous oxide reductase; N_2_, dinitrogen; NarGHI, nitrate reductase; NirBD, cytoplasmic, ammonia-forming nitrite reductase; NrfAH, membrane-bound, ammonia-forming nitrite reductase; NO_2_^−^, nitrite; NH_3_, ammonia

Out of the 24 putative SRB genomes, 14 encoded *mtg* genes, 22 encoded alcohol dehydrogenases, and 22 encoded nickel-iron hydrogenases. All genes annotated as the trimethylamine methyltransferase *mttB* were actually the non-pyrrolysine homolog *mtgB* gene involved in glycine betaine demethylation [56] (Additional file 9: Figure S4). Four MAGs had both subunits B and C encoded adjacently: an *Acidobacteria* (maxbin2.0082), a *Chloroflexi* (maxbin2.0347), and two *Deltaproteobacteria* (maxbin2.0177 and maxbin2.0512). RPKM-based abundances of *mtgB*-containing contigs were significantly higher in wetland P7 (Additional file 9: Figure S4, PERMANOVA, *F* = 4.6677, *p* < 0.001). Three representative genomes are summarized in Fig. 2, and binned *dsrD* genes are specified in the context of their rank abundance in the two wetlands in Additional file 10: Figure S5. Although DsrD taxonomic affiliation was inferred from the best BLASTP hit, bin taxonomy was retrieved from a lineage-specific set of conserved genes via CheckM [26].

Three MAGs (*Chloroflexi*, maxbin2.1011; *Desulfobacteraceae*, metabat2.783; *Nitrospiraceae*, metabat2.164) representing some of the most abundant SRB in both P7 and P8 wetlands encoded remarkably similar and versatile metabolic capabilities (Fig. 2). The complete or almost complete Embden-Meyerhof-Parnas glycolysis pathway and pentose phosphate pathway were present in all three genomes. Besides carbohydrates, other candidate electron donors available to these microorganisms included alcohols (as indicated by the presence of alcohol dehydrogenases), lactate (lactate dehydrogenase), pyruvate (pyruvate water dikinase and pyruvate: ferredoxin oxidoreductase), acetate (acetyl-CoA synthetase), formate (formate dehydrogenase), and hydrogen (nickel-iron hydrogenases). The *Desulfobacteraceae* genome encoded a methanol-specific methyltransferase and the trimethylamine-specific methyltransferase *mttC*, while the *Chloroflexi* genome encoded six *mtgB* genes (Additional file 9: Figure S4). All three genomes encoded the complete or almost complete tricarboxylic acid cycle and the ability to fix carbon dioxide via the Wood-Ljungdahl pathway, which could be reversed to completely oxidize substrates to CO_2_. Respiratory processes included oxygen reduction (evidenced by the presence of a complete electron transport chain: NADH dehydrogenase, succinate dehydrogenase, cytochrome *bd*_*1*_ oxidase, and the *aa*_*3*_-type cytochrome c oxidase in the *Chloroflexi* genome), dissimilatory sulfate reduction (*sat*, *apr*, and *dsrAB*), and dissimilatory nitrate reduction to ammonium (DNRA) via *narGHI*, *nirBD*, and *nrfAH*. The *Chloroflexi* genome also had the potential to perform the last step in denitrification (*nosZ*).

### Candidate methanogens are diverse and may utilize a variety of electron donors

**Fig. 3:**
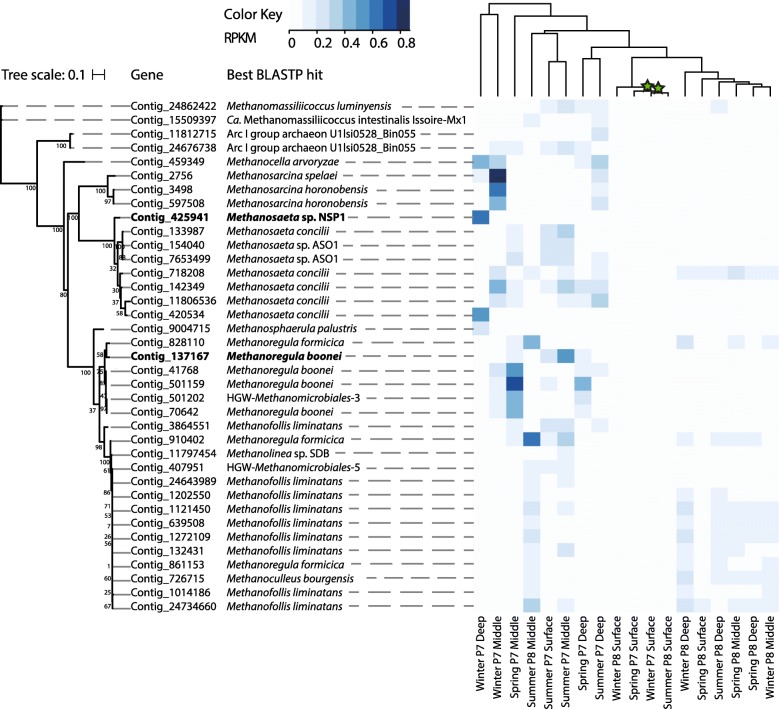
*mcrA* phylogenetic affiliation and abundance per sample. The RAxML tree was constructed using 37 amino acid sequences. The gene affiliation was inferred from the best BLASTP hit. Bolded names represent *mcrA* present in reconstructed genomes. For the heat map, the *mcrA*-containing contig RPKM values were used as input. The statistical significance of hierarchical clustering branches is indicated by green stars (pvclust, approximately unbiased *p* < 0.05)

Thirty-seven *mcrA* sequences affiliated with *Methanofollis* (9), *Methanosaeta* (8), *Methanoregula* (7), *Methanosarcina* (3), Arc I group archaea (2), *Methanomassiliicoccus* (2), HGW *Methanomicrobiales* archaea (2), *Methanocella* (1), *Methanoculleus* (1), *Methanolinea* (1), and *Methanosphaerula* (1) were also recovered from the metagenomic dataset (Fig. 3). Mirroring patterns observed for *dsrD* distributions, *mcrA* gene abundances also differed across the two wetlands (PERMANOVA, *F* = 4.9376, *p* = 0.001), with redundancy analyses confirming that wetland was a primary factor constraining methanogen community structure (Additional file 5: Figure S2). From RPKM values, *mcrA* sequences affiliated with *Methanosaeta concilii* (Contig_718208_1, Contig_142349_4) were inferred to be the most abundant across all samples, followed by *mcrA* genes from *Methanoregula* (Contig_910402_3, Contig_501159_7) and *Methanofollis liminatans* (Contig_24734660_2, Contig_1121450_8) (Fig. 3). Summed *mcrA* RPKM values within the samples indicated that candidate methanogens were most abundant in middle P7 depths (Additional file 7: Table S4). The *mcrA*-based Shannon diversity index varied between 2.25 and 3.3, with no statistical difference between the two wetlands (Additional file 7: Table S4). We also detected three F420-dependent alcohol dehydrogenases (Contig_574620_1, Contig_579739_1, and Contig_24737072_1) with best BLATP hits to *Methanofollis ethanolicus* (WP_067053167.1), but no ribosomal proteins matching this genus.

Two MAGs encoding *mcrA* genes (Contig_425941_8 and Contig_137167_7, respectively) were recovered: a *Methanosaeta* (bin.308) 93.3% complete with 3.27% contamination that was 45 times more abundant in wetland P7 than in P8 and a *Methanoregulaceae* (metabat2.147) 92.68% complete with 15.79% contamination that was 9 times more abundant in P7 sediments than in P8 (Additional file 8: Table S5). Both genomes contained the functional potential for methanogenesis from acetate, formate, and H_2_/CO_2_. Although both acetate kinase and phosphotransacetylase were absent, an acetyl-CoA synthetase (ACSS) and a carbon monoxide dehydrogenase-acetyl-CoA decarbonylase/synthase (CODH/ACDS) were encoded in these genomes. They also encoded a formate dehydrogenase and a formylmethanofuran dehydrogenase. From this point in the pathway, all the genes required for hydrogenotrophic methanogenesis were present in the two genomes: formylmethanofuran-tetrahydromethanopterin *N*-formyltransferase, methenyltetrahydromethanopterin cyclohydrolase, methylenetetrahydromethanopterin dehydrogenase, 5,10-methylenetetrahydromethanopterin reductase, tetrahydromethanopterin S-methyltransferase, methyl-coenzyme M reductase, and heterodisulfide reductase.

### PPR viruses are novel, abundant, and diverse

Viral population abundances and linkages to bacterial hosts were also assessed using the metagenomic data. In total, 3344 viral populations accounting for extensive viral novelty were recovered from the 18 sediment samples. These sequences formed 589 genus-level vContact clusters (Additional file 11: Table S6), with 501 completely new candidate genera (clusters of only PPR sequences), 36 new genera within *Siphoviridae*, 16 within *Podoviridae*, and 14 within *Myoviridae* (within these families, clusters had reference sequences classified only to family level). Reflecting this novelty, only one viral sequence (Contig_372448) had a BLAST hit to the GOV database (GOV_bin_5740_contig-100_7).

The majority of these viral populations (2703 out of 3344) were taxonomically unclassified (Additional file 11: Table S6), while the remainder could be classified as novel or known genera within *Podoviridae* (219), *Myoviridae* (216), *Siphoviridae* (202) and unclassified *Caudovirales* (3) and *Microviridae* (1). Most of these vOTUs (3329) met the criteria to be included in further analyses (see the “Methods” section).

**Fig. 4:**
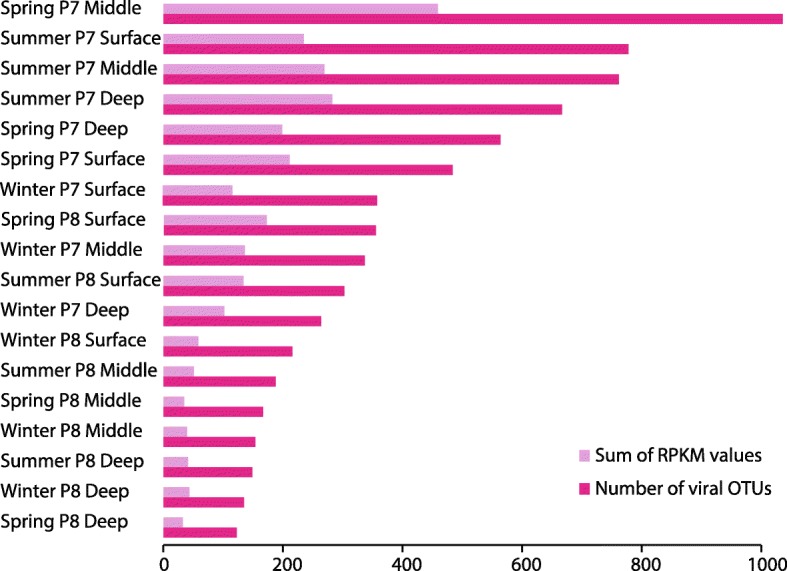
Richness and abundance of viral populations per sample. The *x*-axis displays the number of viral OTUs (darker shade) and abundance (lighter shade) calculated as the sum of viral contig RPKM values in each sample (*y*-axis). Samples are sorted based on decreasing richness

Sediments from wetland P7 collected over spring and summer had the highest numbers of vOTUs and highest total viral abundance (summed RPKM values for all viruses present in that sample). As an example, wetland sediments from P7 at middle depths collected during spring had 1036 vOTUs and a summed RPKM of ~ 459. In contrast, deep sediments collected from wetland P8 at the same time point contained only 123 low abundance vOTUs (summed RPKM = ~ 33) (Fig. 4 and Additional file 7: Table S4). Viral OTU abundances differed significantly between the two wetlands (PERMANOVA, *F* = 5.8165, *p* < 0.001), supporting redundancy analyses of vOTU abundances that identified wetland type as a primary driver of viral community clustering (Additional file 5: Figure S2). Viral Shannon diversity was also higher in P7 (5.9) than in P8 (4.9; paired *t* test, *p* < 0.001; Additional file 7: Table S4).

### Microbial and viral communities correlate

Prior 16S rRNA gene analyses from 215 PPR P7 and P8 wetland sediment samples had identified 1188 OTUs, with each sample harboring ~ 500–700 OTUs [9]. 16S rRNA gene data from the same subset of samples used for metagenomic analyses was re-analyzed here to identify any possible correlation between microbial and viral community structure.

**Fig. 5:**
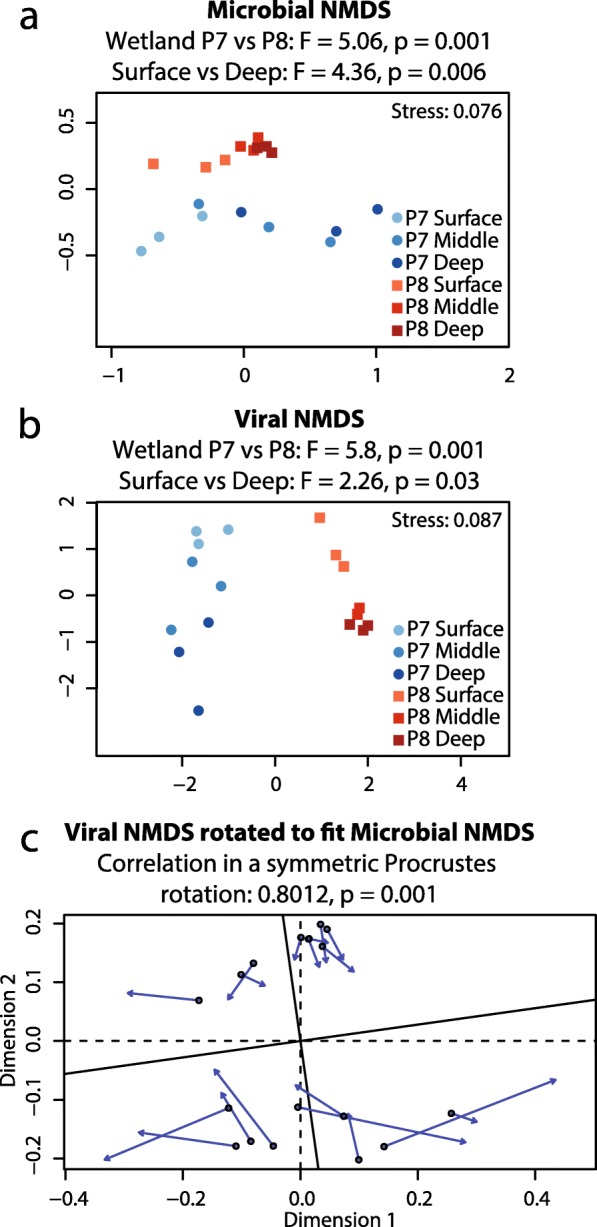
Correlation between microbial and viral populations. **a** 16S rRNA gene-based non-metric multidimensional scaling (NMDS) analyses of microbial community clustering. **b** Viral population-based NMDS. PERMANOVA statistics are provided on top of each plot. Samples were color coded based on significant clustering variables—wetland (P8 in blue and P7 in red) and depth (the deeper, the darker the shade). **c** Procrustes rotation of the viral to the microbial NMDS. Correlation and *p* value are provided on top of the plot

The non-metric multidimensional scaling (NMDS) of 16S rRNA gene data recapitulated overall microbial community trends as previously observed [9], such as strong clustering based on wetland and depth (Fig. 5a). Similar analysis using a RPKM vOTU table for viral diversity and abundance revealed similar clustering trends (Fig. 5b). A strong and significant correlation (0.8, *p* = 0.001) between the viral and the microbial ordinations was identified using a Procrustes rotation (Fig. 5c).

### Viruses can be linked to abundant candidate sulfate reducers and methanogens

**Fig. 6:**
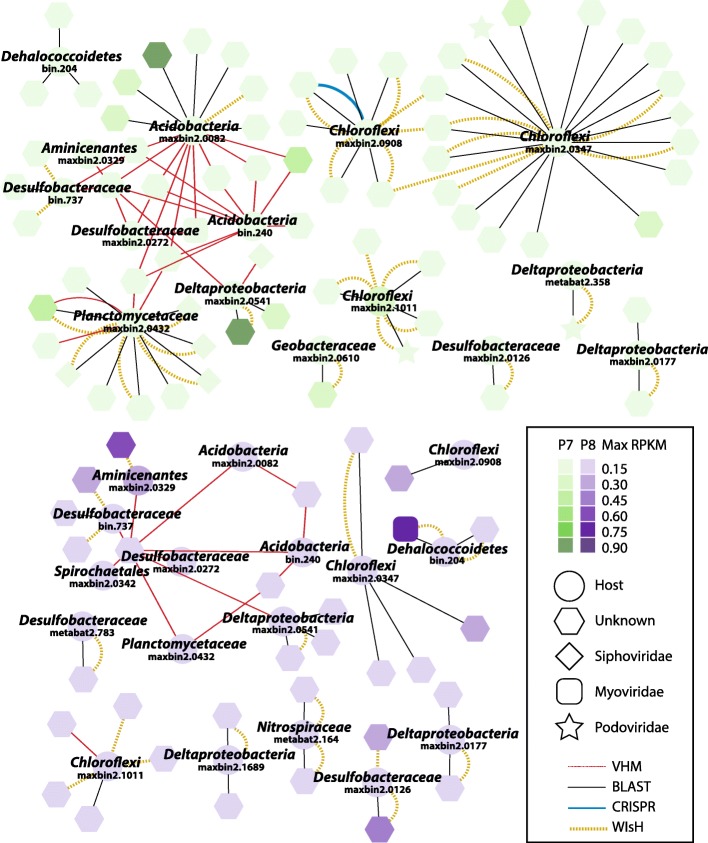
Predicted virus-host linkages among candidate sulfate-reducing strains. Linkages are displayed based on wetland (P7 in green and P8 in purple). Each host (circles) is identified by taxonomic affiliation and genome name, while viruses (other shapes) are only shown based on taxonomy. Increasing abundances are indicated by darker color shades, with abundances represented by average RPKM value across samples from each wetland. For sulfate reducers, the *dsrD*-containing contig was prioritized in RPKM calculations, and only genomes missing *dsrD* had their abundances represented by reductive *dsrA*-containing contigs (Additional file 10: Table S5). The four prediction methods are represented by the different color-coded lines

Four methods were used to identify viruses that could infect candidate SRB and methanogen hosts: matches between CRISPR spacers and viral contigs, blasting viral contigs to microbial genomes in order to find prophages, and two *k*-mer frequency-based prediction tools (VirHostMatcher and WIsH). The results for SRB hosts are summarized in Fig. 6, which displays both the number of links and the abundance of hosts and viruses across the two wetlands. While similar numbers of SRB hosts could be linked to viruses in P7 (15) and P8 (17), the overall number of virus-host linkages (pairs) was larger in P7 (88) than in P8 (40). The predicted hosts included some of the most abundant sulfate reducers in each wetland: two *Chloroflexi* in wetland P7 (maxbin2.1011 and maxbin2.0347) and strains associated with Candidatus *Aminicenantes* (maxbin2.0329), *Desulfobactereaceae* (metabat2.783), and *Nitrospirae* (metabat2.164) in wetland P8. Most of the individual links (69) occurred via BLAST, with 40 via WIsH, 27 via VirHostMatcher, and only 1 via CRISPR spacer matching. Finally, the methanogen *Methanosaeta* MAG was tentatively linked to two viral contigs (Contig_425558 and Contig_425713) via WIsH.

## Discussion

This study aimed to investigate the diversity and metabolic potential of sulfate-reducing microorganisms, methanogens, and viruses in PPR wetland sediments that could contribute to, or impact, the highest sulfate reduction rates ever measured as well as some of the highest methane emissions from wetlands in North America [9]. Reflecting the range of carbon substrates detected in PPR sediment pore fluids, diverse communities of metabolically flexible SRB and methanogens were identified that could potentially drive high rates of biogeochemical transformations.

### Sulfate reduction is likely performed by diverse, metabolically flexible microorganisms

Diverse putative SRB were identified in PPR sediments via both metagenomic screening of marker genes (162 *dsrA* and 206 *dsrD* sequences) (Fig. 1, Additional file 4: Figure S1, Additional file 10: Figure S5, Additional file 3: Table S3) and genome-resolved metagenomics that enabled recovery of 24 inferred SRB genomes that span seven phyla (Additional file 8: Table S5). These genomes should be considered to represent candidate sulfate reducers, given that genomic information cannot guarantee the direction of the reaction, as previously shown by the discovery that the sulfur-oxidizing microorganism *D. alkaliphilus* encodes a reductive-type dissimilatory sulfite reductase [55]. Moreover, one genome (bin.240) in this study encoded only *dsrD* and no other *dsr* genes, and another (maxbin2.0329) encoded only *dsrD* and *dsrC*. While this may be due to genome completeness limitations (Additional file 8: Table S5), we could not clearly determine the potential for sulfate reduction in these cases. Future isolation of these microorganisms is required to confirm sulfate reduction.

These genomes revealed a high level of metabolic flexibility, through the potential utilization of a variety of electron donors and acceptors. We have previously identified a wide diversity of electron donors in PPR pore fluids, including micromolar concentrations of acetate and methanol and millimolar concentrations of ethanol and 2-propanol [9]. The metabolic potential for utilization of such substrates in SRB MAGs strengthens the hypothesis that these carbon pools could support the measured SRRs. In particular, C1 substrates may play an important role in sustaining sulfate reduction in this system. One candidate SRB MAG encoded a methanol dehydrogenase, while two other MAGs encoded *mtaA*, a methanol-specific methyltransferase. Souza et al. previously identified two methanol degradation pathways in the sulfate reducer *Desulfotomaculum kuznetsovii*: one via alcohol dehydrogenase and one via methyltransferases *mtaABC* [57], while methanol oxidation via a methyltransferase system has also been described in *Sporomusa* species [58]. Arshad et al. also identified methanol and methylamine methyltransferases in the genome of *Candidatus* Nitrobium versatile [59], a candidate sulfate reducer that also encoded versatile metabolic potential remarkably similar to the genomes recovered in this study, including the *Nitrospiraceae* MAG (Fig. 2). The potential for metabolism of methylamines was also present in inferred sulfate reducer MAGs recovered in this study; two MAGs encoded *mtb* genes (Additional file 9: Figure S4 and Additional file 8: Table S5). The non-pyrrolysine *mttB* homolog methyltransferase *mtgB* present in 14 of our candidate sulfate reducer genomes has previously been shown to allow utilization of glycine betaine as an electron donor in *Desulfitobacterium hafniense* [56], *Sporomusa ovata* [60], and, potentially, *Candidatus* Frackibacter [34]. These data again highlight the metabolic diversity within the pool of putative SRB in this system and suggest that C1 metabolism may be a more widespread characteristic of SRB than currently appreciated.

Additional metabolic diversity associated with electron acceptor utilization was identified within the same MAGs and could allow SRB to respond to dynamic environmental conditions in near-surface wetland sediments that may be exposed to oxygen, inputs of nitrogen species from adjacent agricultural regions, and fluctuations in redox. These inferred traits may represent another mechanism that at least partially explains the high SRRs in this system. Finally, the phylogenetic and functional diversity of SRB within this system may support a high degree of niche differentiation within the geochemically heterogeneous sediment matrix [61–64], allowing a variety of sulfate-reducing groups to perform sulfate reduction concomitantly and thus increase overall sulfate reduction rates.

The application of genome-resolved metagenomics to sulfate-reducing microbial communities has recently identified this functional trait in a wide range of microbial taxa not previously thought to catalyze this reaction [54, 65, 66]. Results from this study—identifying the potential for sulfate reduction in *Acidobacteria*, *Armatimonadetes*, *Planctomycetes*, and *Candidatus* Schekmanbacteria—support the results from Anantharaman et al. [54] and suggest that additional SRB diversity remains to be uncovered. This is the first study to report *dsrD* in the members of the candidate phylum *Aminicenantes* (former OP8). The *Aminicenantes* MAG reconstructed here was only ~ 50% complete and also encoded *dsrC*, but lacked *dsrAB*; therefore, it remains unclear whether this organism could perform sulfate reduction. However, the *Aminicenantes dsrC* had both C-terminal conserved cysteine residues [67] and its *dsrD* was the most abundant binned *dsrD* gene in wetland P8, suggesting that this organism was playing an active role in community functioning. The high relative abundances of these newly identified, putative SRB lineages in PPR sediments (Fig. 1 and Additional file 4: Figure S1) suggest that they may play a role in driving the extremely high SRRs and may contribute to the rate differences between wetlands. Prior 16S rRNA gene analyses had highlighted the contribution of OTUs matching poorly resolved *Chloroflexi*, *Deltaproteobacteria*, *Actinobacteria*, and *Acidobacteria* to the Bray-Curtis dissimilarity between P7 and P8 [9]. Although putative SRB diversity measured using Shannon’s diversity index was similar between wetlands, differential *dsrD* abundances affiliated with these taxa (Additional file 10: Figure S5) suggest that community membership and structure, in addition to activity, may be a factor contributing to the higher measured SRRs in wetland P7.

### A variety of electron donors could fuel methanogenesis in PPR sediments

Concurrent with high rates of sulfate reduction, we have previously measured extremely high methane fluxes from these small prairie wetlands. We recovered 37 *mcrA* sequences affiliated with the orders *Methanomicrobiales* (*Methanosphaerula*, *Methanolinea*, *Methanoregula*, *Methanoculleus*, and *Methanofollis* and HGW lineages [68]), *Methanosarcinales* (*Methanosaeta* and *Methanosarcina*), *Methanocellales*, *Methanomassiliicoccales*, and the *Methanomicrobia* Arc I group archaea from the metagenomic data and were able to assemble two MAGs that were taxonomically classified as *Methanosaeta* and *Methanoregulaceae.* These two MAGs represented the two most abundant taxa in sampled sediments. Typically, *Methanosaeta* produce methane from acetate [69], while *Methanoregulaceae* utilize formate or H_2_/CO_2_ for methanogenesis [70]. These genomes both encoded ACSS, CODH/ACDS, formate dehydrogenase, and all the core genes in the hydrogenotrophic pathway. Given that acetoclastic methanogenesis has not been previously reported in this family, *Methanoregulaceae* likely require the ACSS gene for biomass synthesis from acetate.

Wetland type again exerted control on abundances of inferred methanogens. Methanogen *mcrA* sequences were more abundant in wetland P7 (Additional file 7: Table S4), where higher pore water methane concentrations (up to 6 mM) were detected [9], and were affiliated with *Methanosarcina*, *Methanosaeta*, and *Methanoregula* (Fig. 3). In contrast, *Methanofollis*-affiliated *mcrA* sequences were more abundant in wetland P8 sediments that generally contained lower pore water methane concentrations (up to 4 mM).

Mirroring the sulfate-reducing populations, the diversity of detected methanogens suggests that a wide range of substrates—including acetate, hydrogen and formate, C1 compounds, and primary and secondary alcohols—could potentially be utilized for methanogenesis. While Arc I group archaea have been hypothesized to produce methane from methylated thiol groups [71], *Methanosarcina* species can utilize H_2_/CO_2_, acetate, dimethylsulfide, methanol, monomethylamine, dimethylamine, and trimethylamine [72, 73], and *Methanomassiliicoccus luminyensis* is able to grow on methanol, mono-, di-, or trimethylamine with hydrogen [74]. Moreover, *Methanofollis ethanolicus* can utilize ethanol/CO_2_, 1-propanol/CO_2_, 1-butanol/CO_2_, H_2_/CO_2_, and formate for growth and methane production, converting ethanol to methane and acetate [75], while *Methanofollis liminatans* can utilize formate, H_2_/CO_2_, 2-propanol/CO_2_, 2-butanol/CO_2_, and cyclopentanol/CO_2_, converting these secondary and cyclic alcohols to their respective ketones [76].

Given the prior measurements of high concentrations of ethanol and 2-propanol in PPR pore fluids (up to 4 mM), the abundance of alcohol-utilizing *Methanofollis* species (best BLASTP hit for 9 of 37 *mcrA* sequences and RPKM values) indicates that these alcohols may fuel methanogenesis in PPR wetlands. Supporting this hypothesis, three F420-dependent alcohol dehydrogenase sequences with best BLASTP hits to *Methanofollis* were detected within the metagenomic data. The absence of ribosomal proteins affiliated to this genus in our dataset suggests that some alcohol-utilizing methanogens in this study may be only distantly related to *Methanofollis*.

### Local geochemistry exerts a strong control on microbial and viral community composition and structure

The community clustering of particular microbial groups (sulfate reducers and methanogens), whole microbial communities, or whole viral communities was primarily based on wetland. (Additional file 5: Figure S2). Furthermore, a strong correlation was measured between microbial and viral communities (Fig. 5) that likely reflects host availability and different microbial community structures in the two wetlands. Despite being only ~ 350 m apart, the P7 and P8 wetlands are characterized by distinct geochemical profiles associated with local hydrology and evapotranspiration processes (Additional file 12: Figure S6) [77–79]. While P8 pore waters contain higher concentrations of sulfate and sulfide, similar fluids from P7 sediments generally contain higher pore water concentrations of methane, ferrous iron, acetate, acetone, methanol, ethanol, and 2-propanol [9]. The trends observed in this study highlight the heterogeneity of geochemical and microbial parameters over short spatial scales in PPR wetlands and demonstrate that strong geochemical controls on microbial and viral community composition and structure can differentially impact the ecosystem functions such as sulfate reduction rates and methane fluxes.

### Novel and abundant viruses may impact carbon and sulfur cycling

A large number of diverse, novel viral populations were identified within this dataset. Given that this is only the second study to investigate viral sequences from wetland sediment metagenomes [80], this novelty is expected and is reflected in the fact that almost no viral contigs from our data were identified in publicly available viral databases, and ~ 80% could not be assigned to any known taxonomic level. These data thus contribute to exploring the under-sampled soil virosphere; despite the estimate that 97% of viruses on Earth are in soils and sediments, as of 2016, only 2.5% of publicly available viromes were from these ecosystems [81].

Viral abundance, richness, and Shannon diversity were significantly higher in P7 wetland samples that also hosted higher rates of microbial activity (as inferred from SRRs) (Fig. 4). While this may simply reflect differences in microbial community composition and structure across the two wetlands, it has previously been suggested that higher host metabolic activity (growth rates on different electron donors) will be associated with higher viral production [82]. This correlation has been observed by Pan et al., who reported significant correlations between viral productivity and microbial metabolism inferred from acetate consumption and CO_2_ production in amended sediment slurries under nitrate-reducing conditions [83]. Recent studies have also suggested that dissolved organic matter (DOM) may impact the rates of viral infection and cell lysis, although a mechanism has yet to be elucidated [14, 84, 85]. Such interactions may be prevalent across PPR wetland ecosystems given the high DOM concentrations frequently measured in pore fluids. Future studies on viral productivity are needed to uncover the dynamics of viral and host activities in PPR wetland sediments.

Our results also highlighted specific viruses predicted to infect the most abundant candidate SRB and methanogens in PPR wetland sediments. Surprisingly, some viruses were predicted to target microorganisms across different phyla, particularly using the VirHostMatcher method. Although we used a stringent threshold (*d*_2_^*^ < 0.2) for inferring viral-host linkages, it is possible that those predictions are false positives. Nonetheless, Peters et al. have isolated phages that infect different taxonomic orders [86], and Paez-Espino et al. have observed CRISPR spacer matches across different phyla [87]. Therefore, at this stage, we could not rule out the possibility that such linkages in these data reflect phages with exceptionally broad host range.

The impacts of viral predation on these microorganisms at the ecosystem function level remain to be elucidated. It is possible that through the infection and lysis of bacterial hosts, viruses could decrease the activity of fast-growing microorganisms [14, 15], potentially repressing sulfate reduction (and associated carbon mineralization) and methane production. Alternatively, the release of labile intracellular contents following virus-mediated cell death may stimulate activity of other microbial community members [81, 88], increasing net sulfate reduction and methane production rates. Given that bacterial cell lysis may open new niche space within the ecosystem, the availability of freshly released labile carbon may also increase microbial diversity in the environment [89]. Additional laboratory experiments with enrichments and even isolated cultures are needed, coupled with these field observations, to better understand how viral predation affects the rates of sulfate reduction and methanogenesis in these wetlands.

## Conclusions

Our results indicate that phylogenetically diverse sulfate-reducing bacteria (SRB) and methanogens are the keys to driving rapid carbon and sulfur transformations in PPR wetland sediments. Candidate SRB identified in this study spanned ten phyla, with some affiliating to taxa only recently described as potential sulfate reducers (*Acidobacteria*, *Armatimonadetes*, *Planctomycetes*, *Candidatus* Schekmanbacteria, and *Gemmatimonadetes*) or that had not been previously described as such (*Aminicenantes*). Candidate methanogens are affiliated to five orders, with particularly abundant sequences related to the genera *Methanosaeta*, *Methanoregula*, and *Methanofollis*. Recovered SRB MAGs encoded versatile metabolic potential, likely reflecting adaptations to dynamic geochemical conditions in the shallow wetland sediments. Based on the metabolic potential encoded in draft genomes, marker gene analyses, and available candidate substrates, a variety of electron donors (i.e., methylamines, methanol, ethanol, 2-propanol, acetate, formate, hydrogen/CO_2_) could fuel sulfate reduction and methanogenesis in this system. Given the abundance of *Methanofollis*-related sequences and previously measured millimolar concentrations of ethanol and 2-propanol in sediment pore fluids [9], we hypothesize these alcohols may drive a significant proportion of methanogenesis in this system. Moreover, SRB genomes encoded genes for the utilization of methanol, methylamines, and glycine betaine as electron donors, suggesting that C1 metabolism may play a significant role in driving high sulfate reduction rates. Abundant viral populations were identified, with a phylogenetic diversity and novelty expected given the scarcity of viral sequences from sediments in databases. These viral populations were predicted to target abundant SRB and methanogens, thus likely impacting carbon and sulfur cycling. While these impacts remain to be elucidated in future studies, this work highlights that a combination of phylogenetic and metabolic diversity controlled by local geochemistry and, potentially, viruses, may explain extremely high methane emissions and sulfate reduction rates in PPR wetlands.

## Additional files

Additional file 1: **Table S1.** Summary of samples. Sample names in this study are corresponded to samples names in our previous 16S rRNA gene study [9]. Genomic DNA (gDNA) concentrations obtained for each sample and total DNA retrieved are presented. (XLSX 10 kb)

Additional file 2: **Table S2.** Assembly statistics. A summary of assembly statics (number of mapped reads and contig length) is provided for both each individual assembly and the merged contig set. (XLSX 13 kb)

Additional file 3: **Table S3.** RPKM values for viral, reductive *dsrA*-, *dsrD*-, and *mcrA*-containing contigs. These tables were used as inputs for many of the analyses indicated in the Methods session. (XLSX 376 kb)

Additional file 4: **Figure S1.** *dsrD* phylogenetic affiliation and abundance per sample. This is the expanded version of Fig. 1. The RAxML tree was constructed using 206 amino acid sequences. The gene affiliation was inferred from the best BLASTP hit. The 23 clusters in Fig. 1 are indicated here. Bolded names represent *dsrD* present in reconstructed genomes. The yellow, blue, and orange stars indicate *dsrD* in genomes represented in Fig. 2. For the heat map, *dsrD*-containing contig RPKM values were used as input. The statistical significance of hierarchical clustering branches is indicated by green stars (pvclust, approximately unbiased *p* < 0.05). (PDF 1128 kb)

Additional file 5: **Figure S2.** Redundancy analyses (RDA) of microbial and viral populations. Each gene abundance (contig RPKM value) was used as input for RDA. The genes reductive *dsrA* and *dsrD* represent candidate sulfate-reducing populations, while *mcrA*, candidate methanogens. Forward selection provided the variables to constrain these populations, shown in the plots and stated below each plot with their associated RDA statistics. In all plots, P7 samples are indicated by gray circles, while P8 samples by white/empty circles. (PDF 518 kb)

Additional file 6: **Figure S3.** *dsrA* phylogenetic affiliation and abundance per sample. The RAxML tree was constructed using 162 amino acid sequences. The gene affiliation was inferred from the best BLASTP hit. For the heat map, the *dsrA*-containing contig RPKM values were used as input. The statistical significance of hierarchical clustering branches is indicated by green stars (pvclust, approximately unbiased *p* < 0.05). (PDF 391 kb)

Additional file 7: **Table S4.** Summary of RPKM values, number of viral OTUs, and Shannon diversity index. A per sample summary of these values is provided. (XLSX 13 kb)

Additional file 8: **Table S5.** Summary of microbial genomes. This table provides a summary of marker genes, completeness, contamination, and RPKM values for genomes investigated in this study. (XLSX 16 kb)

Additional file 9: **Figure S4.** Analyses of MtgB genes found in candidate sulfate reducer genomes. **a**. This RAxML tree displays the trimethylamine: corrinoid methyltransferase MttB and the affiliation of MtgB sequences from this study (in black). Pyrrolysine-containing reference sequences are shown in orange, and non-pyrrolysine-containing reference sequences are shown in blue. Reference sequences were retrieved from Daly et al. and Ticak et al. [34, 56]. **b**. Analyses of *mtgB* abundances. The RAxML tree was constructed using 28 amino acid sequences. The gene affiliation was inferred from the best BLASTP hit. All sequences were present in reconstructed genomes. For the heat map, the *mtgB*-containing contig RPKM values were used as input. The statistical significance of hierarchical clustering branches is indicated by green stars (pvclust, approximately unbiased *p* < 0.05). (PDF 311 kb)

Additional file 10: **Figure S5.** *dsrD* rank abundance curves in P7 and P8. The average RPKM value of *dsrD*-containing contigs in each wetland is displayed in the *y*-axis, while each one of the 206 genes is in the *x*-axis. Sequences present in genomes are indicated by different colors, with the genome taxonomic affiliation and name indicated. (PDF 386 kb)

Additional file 11: **Table S6.** Summary of viral taxonomy. Taxonomic classification and vContact-based clustering for each viral contig are provided. (XLSX 81kb)

Additional file 12: **Figure S6.** Principal component analysis (PCA) of geochemical variables. Pore water concentrations of sulfate, sulfide, ferrous iron (Fe II), and methane were retrieved from Dalcin Martins et al. [9] and used as input values for this analysis. P7 samples are represented by black circles, while P8 samples by gray circles. (PDF 102 kb)

## Abbreviations

CH_4_: Methane
CO_2_: Carbon dioxide
CODH/ACDS: Carbon monoxide dehydrogenase-acetyl-CoA decarbonylase/synthase
DOM: Dissolved organic matter
GOV: Global Ocean Virome
H_2_: Hydrogen
HMM: Hidden Markov model
MAG: Metagenome-assembled genome
NMDS: Non-metric multidimensional scaling
OTU: Operational taxonomical unit
PPR: Prairie Pothole Region
RDA: Redundancy analyses
RPKM: Reads Per Kilobase per Million mapped reads
SRB: Sulfate-reducing bacteria
SRR: Sulfate reduction rate
vOTU: Viral operational taxonomical unit
