# Supplementary figures and images for: Viral and metabolic controls on high rates of microbial sulfur and carbon cycling in wetland ecosystems
Source: Microbiome. 2018 Aug 7;6:138. doi: 10.1186/s40168-018-0522-4 (PMC6081815; doi:10.1186/s40168-018-0522-4)

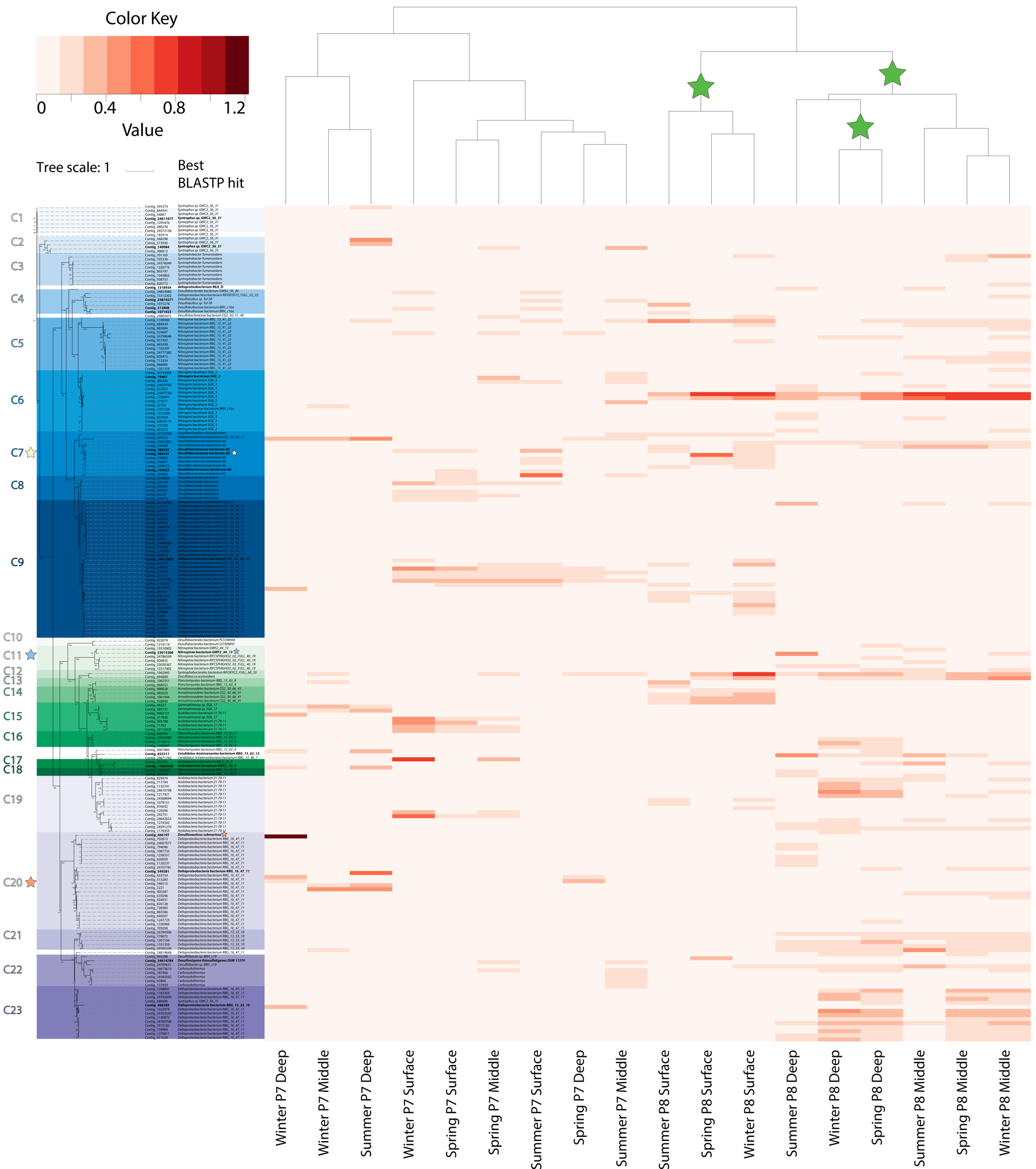

Supplement: Supplementary file 4 — Figure S1. dsrD phylogenetic affiliation and abundance per sample. This is the expanded version of Fig. 1. The RAxML tree was constructed using 206 amino acid sequences. The gene affiliation was inferred from the best BLASTP hit. The 23 clusters in Fig. 1 are indicated here. Bolded names represent dsrD present in reconstructed genomes. The yellow, blue, and orange stars indicate dsrD in genomes represented in Fig. 2. For the heat map, dsrD-containing contig RPKM values were used as input. The statistical significance of hierarchical clustering branches is indicated by green stars (pvclust, approximately unbiased p < 0.05). (PDF 1128 kb) [file 40168_2018_522_MOESM4_ESM.pdf]

*dsrD*

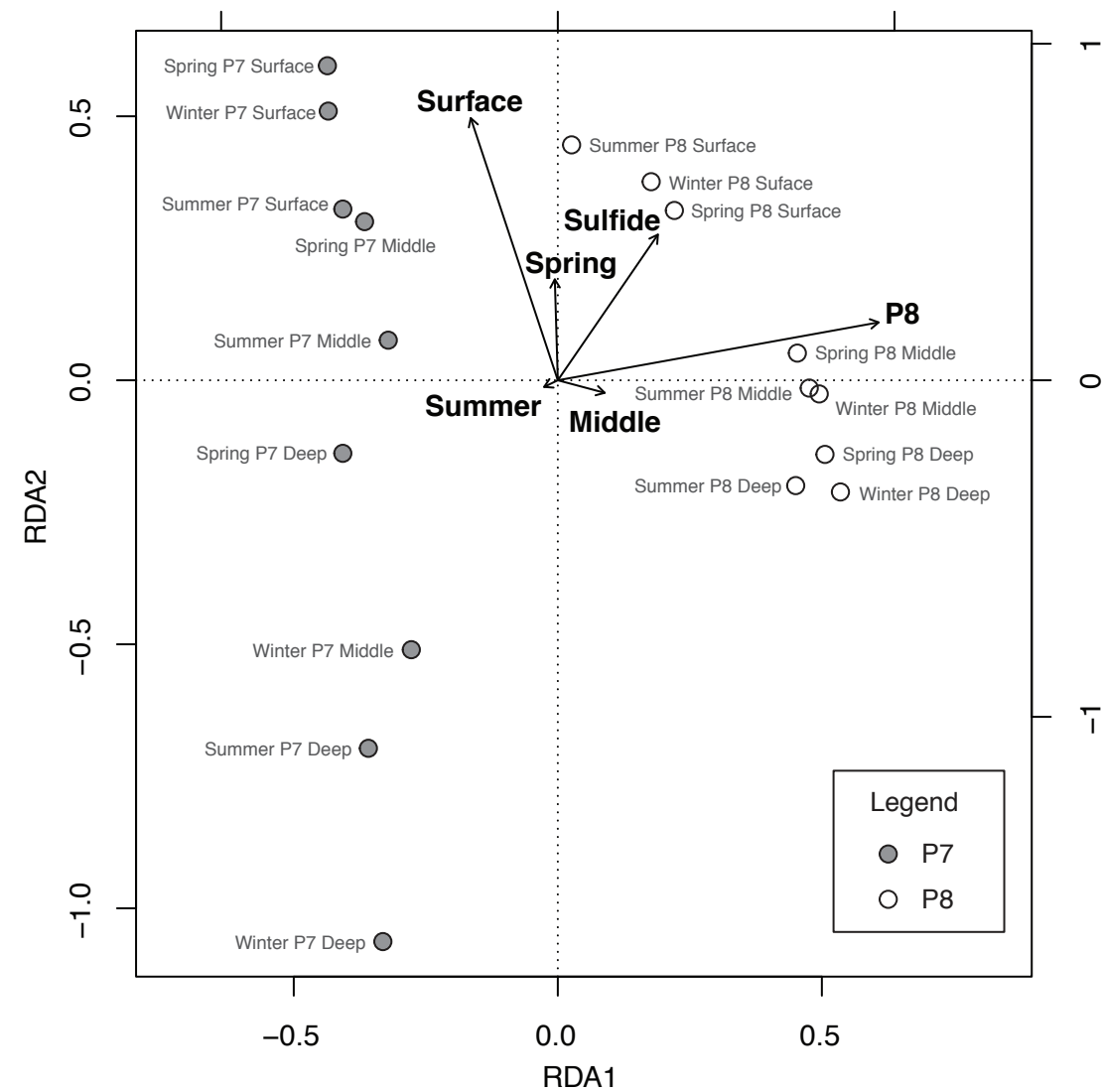

*dsrA*

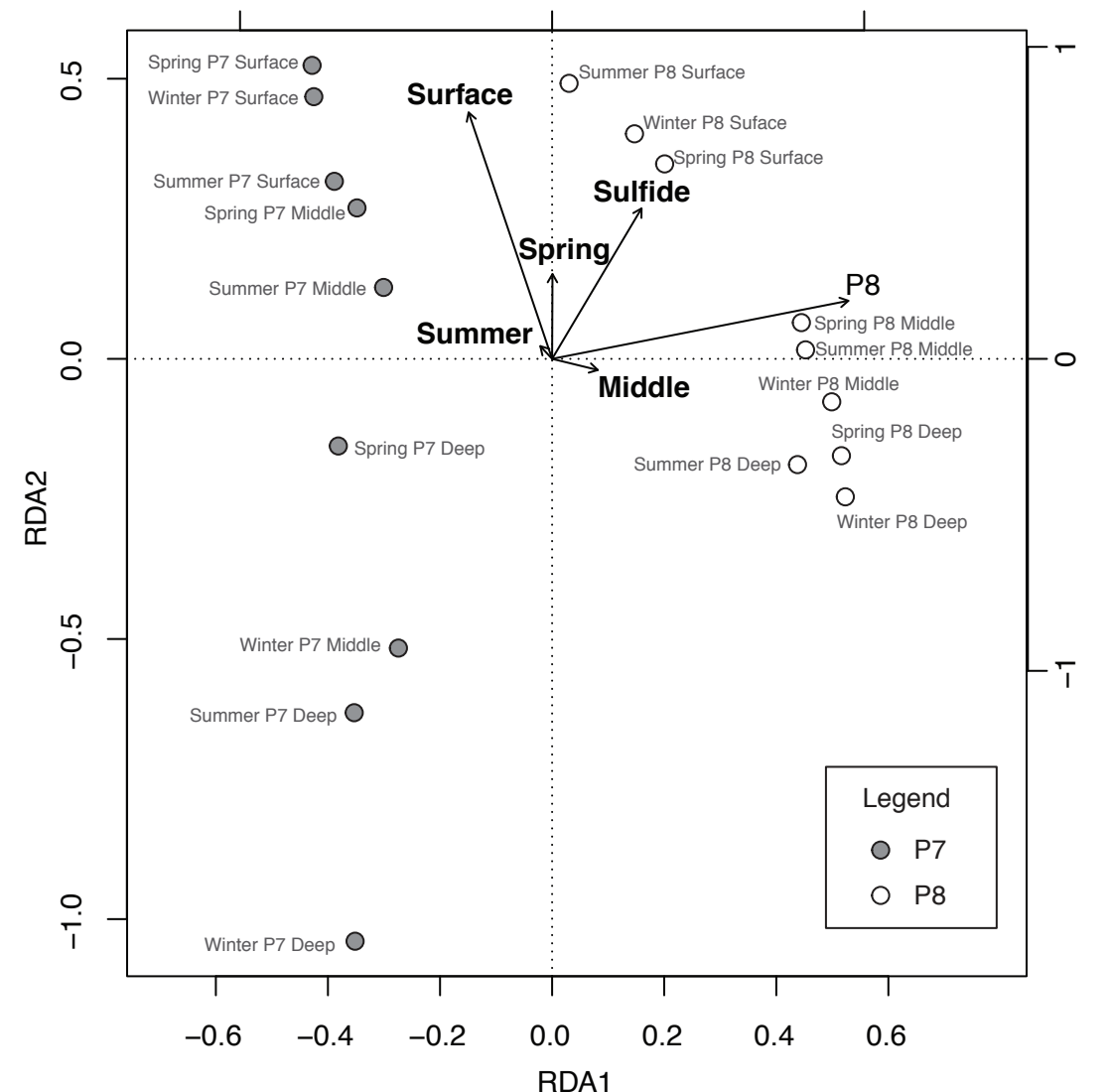

*mcrA*

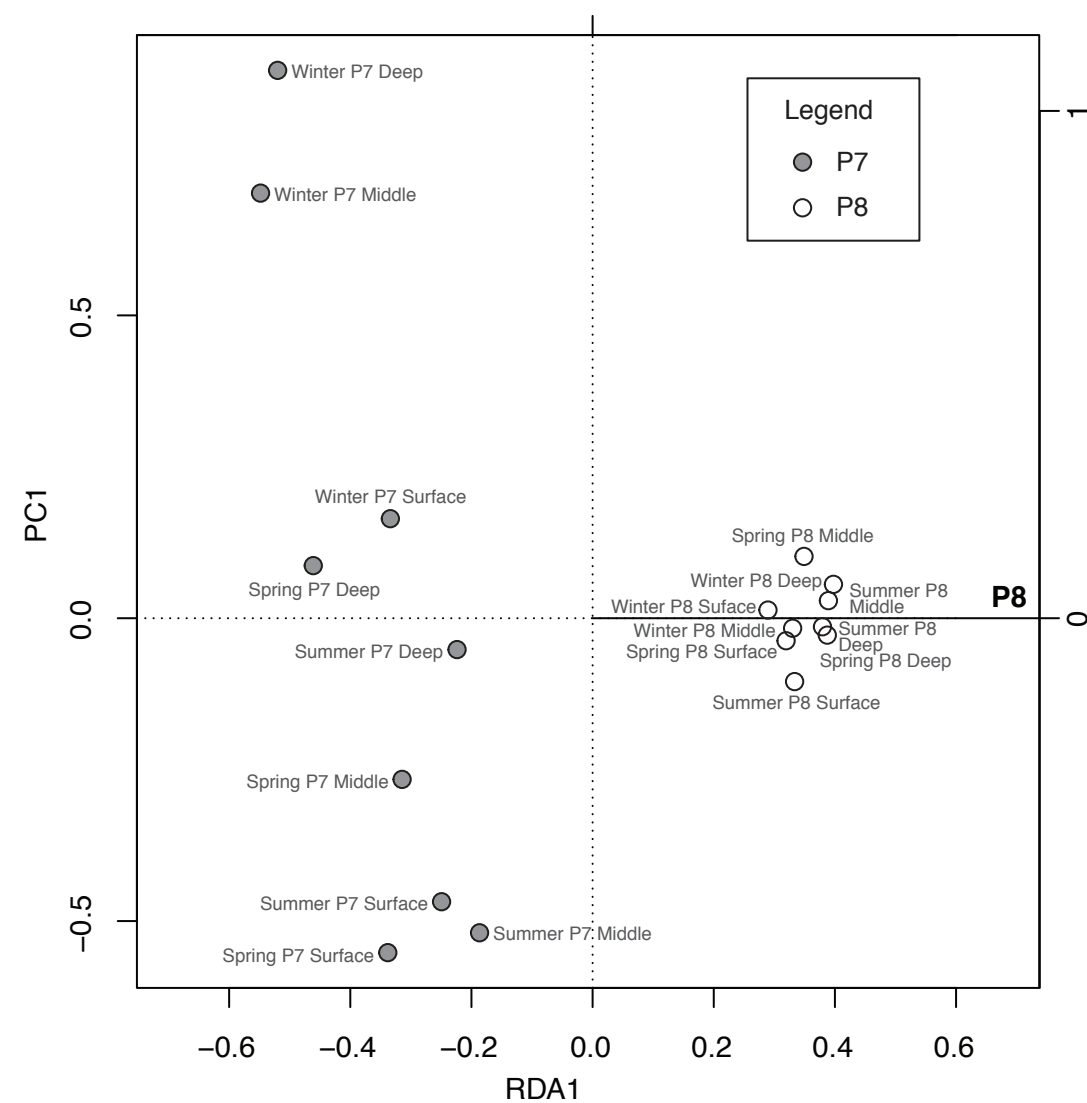

viruses

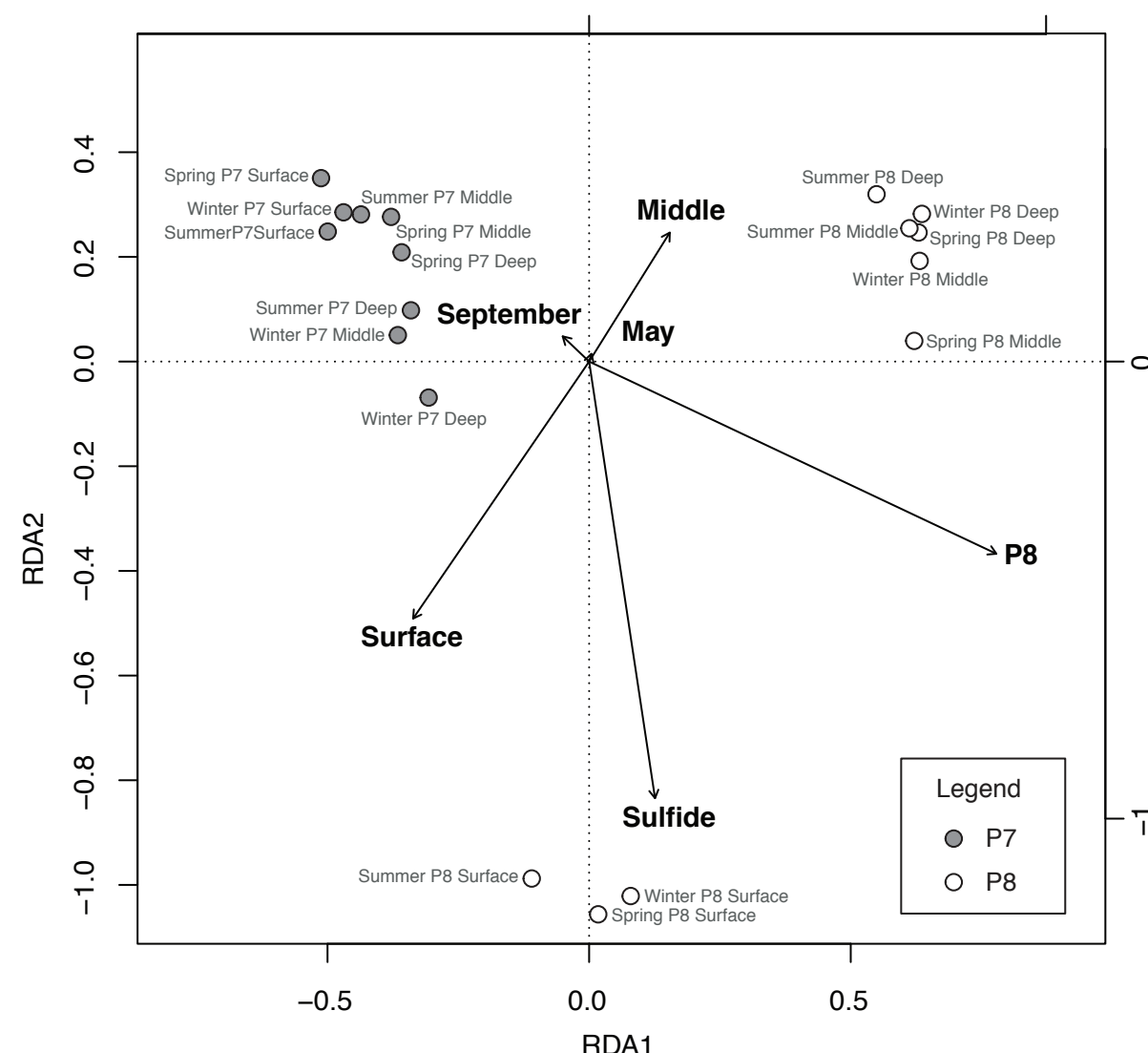

Supplement: Supplementary file 5 — Figure S2. Redundancy analyses (RDA) of microbial and viral populations. Each gene abundance (contig RPKM value) was used as input for RDA. The genes reductive dsrA and dsrD represent candidate sulfate-reducing populations, while mcrA, candidate methanogens. Forward selection provided the variables to constrain these populations, shown in the plots and stated below each plot with their associated RDA statistics. In all plots, P7 samples are indicated by gray circles, while P8 samples by white/empty circles. (PDF 518 kb) [file 40168_2018_522_MOESM5_ESM.pdf]

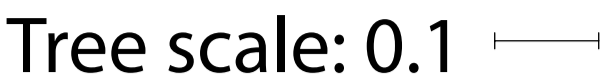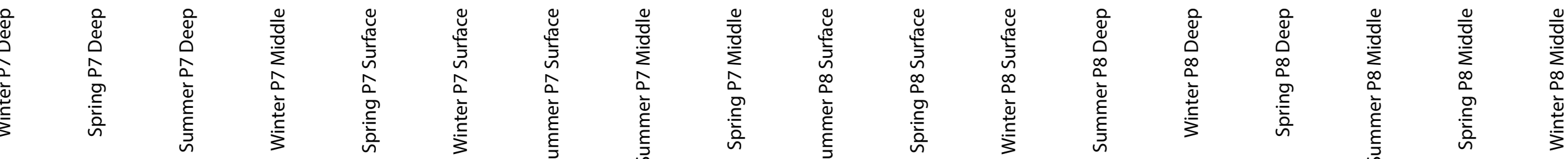

Supplement: Supplementary file 6 — Figure S3. dsrA phylogenetic affiliation and abundance per sample. The RAxML tree was constructed using 162 amino acid sequences. The gene affiliation was inferred from the best BLASTP hit. For the heat map, the dsrA-containing contig RPKM values were used as input. The statistical significance of hierarchical clustering branches is indicated by green stars (pvclust, approximately unbiased p < 0.05). (PDF 391 kb) [file 40168_2018_522_MOESM6_ESM.pdf]

Average RPKM

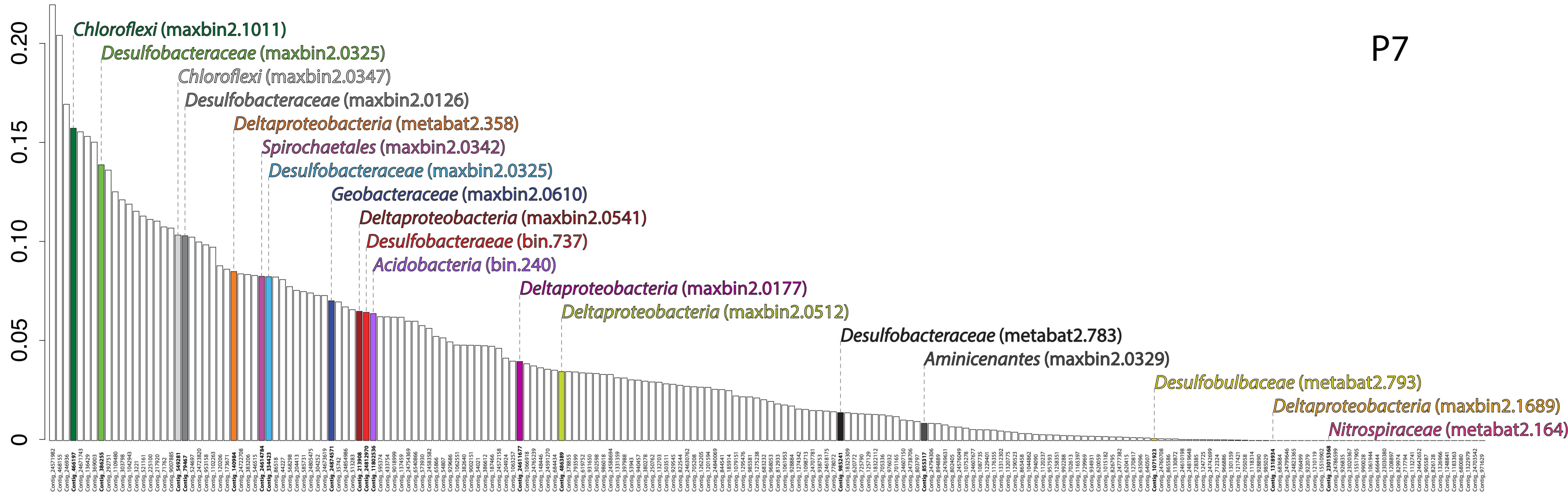

P7

Average RPKM

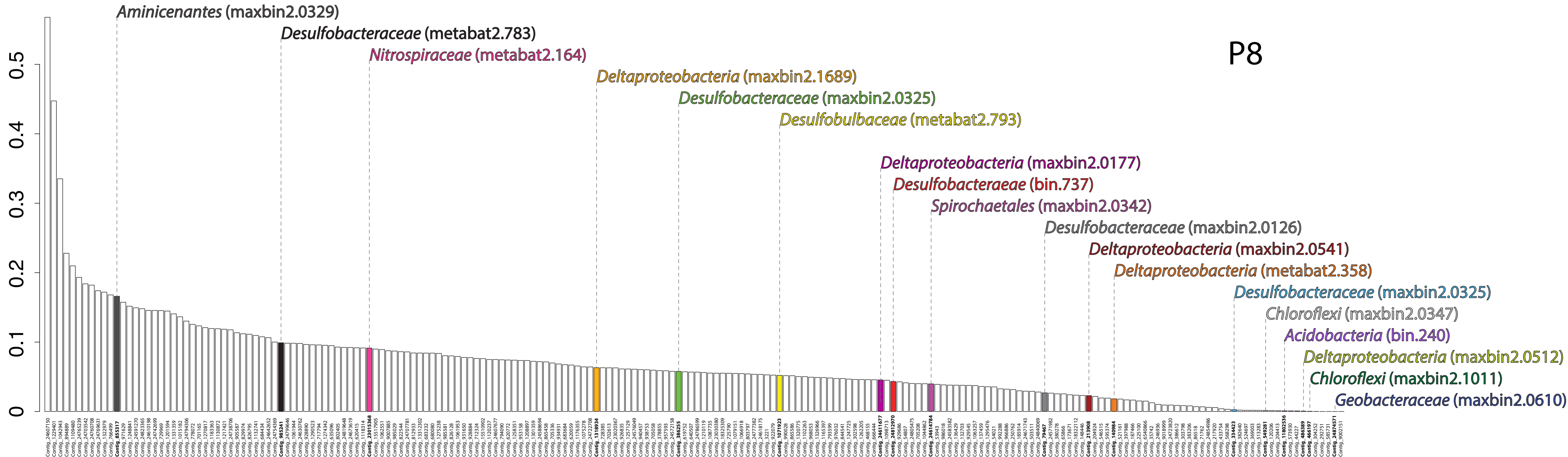

P8

Supplement: Supplementary file 10 — Figure S5. dsrD rank abundance curves in P7 and P8. The average RPKM value of dsrD-containing contigs in each wetland is displayed in the y-axis, while each one of the 206 genes is in the x-axis. Sequences present in genomes are indicated by different colors, with the genome taxonomic affiliation and name indicated. (PDF 386 kb) [file 40168_2018_522_MOESM10_ESM.pdf]

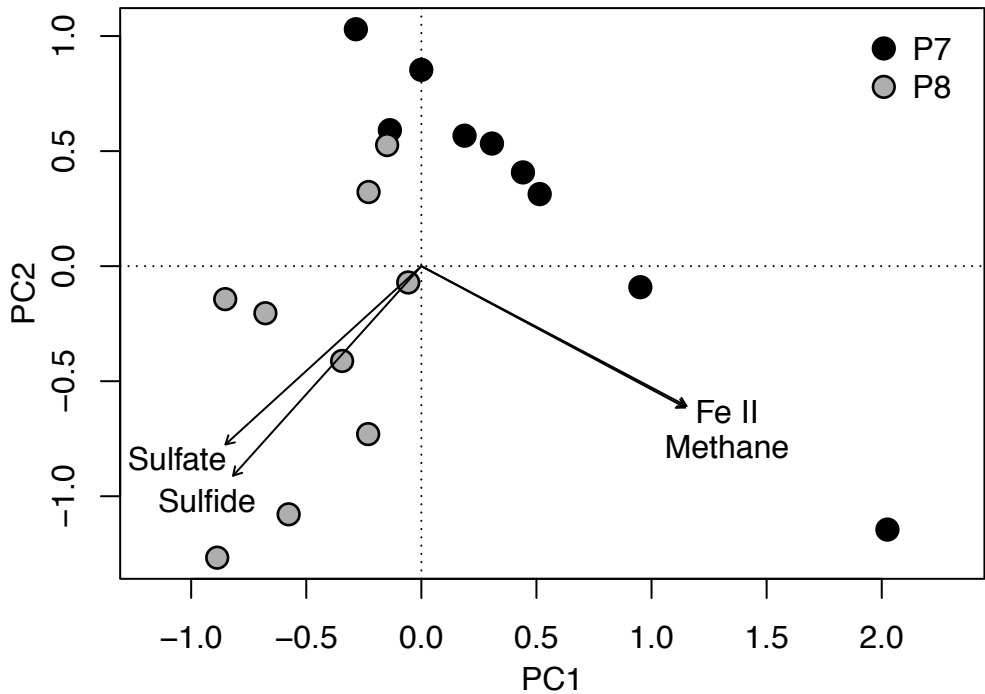

Supplement: Supplementary file 12 — Figure S6. Principal component analysis (PCA) of geochemical variables. Pore water concentrations of sulfate, sulfide, ferrous iron (Fe II), and methane were retrieved from Dalcin Martins et al. [9] and used as input values for this analysis. P7 samples are represented by black circles, while P8 samples by gray circles. (PDF 102 kb) [file 40168_2018_522_MOESM12_ESM.pdf]
