# Supplementary material for: Viral and metabolic controls on high rates of microbial sulfur and carbon cycling in wetland ecosystems
Source: Microbiome. 2018 Aug 7;6:138. doi: 10.1186/s40168-018-0522-4 (PMC6081815; doi:10.1186/s40168-018-0522-4)
Supplement: Supplementary file 9 — Figure S4. Analyses of MtgB genes found in candidate sulfate reducer genomes. a. This RAxML tree displays the trimethylamine: corrinoid methyltransferase MttB and the affiliation of MtgB sequences from this study (in black). Pyrrolysine-containing reference sequences are shown in orange, and non-pyrrolysine-containing reference sequences are shown in blue. Reference sequences were retrieved from Daly et al. and Ticak et al. [34, 56]. b. Analyses of mtgB abundances. The RAxML tree was constructed using 28 amino acid sequences. The gene affiliation was inferred from the best BLASTP hit. All sequences were present in reconstructed genomes. For the heat map, the mtgB-containing contig RPKM values were used as input. The statistical significance of hierarchical clustering branches is indicated by green stars (pvclust, approximately unbiased p < 0.05). (PDF 311 kb) [file 40168_2018_522_MOESM9_ESM.pdf]

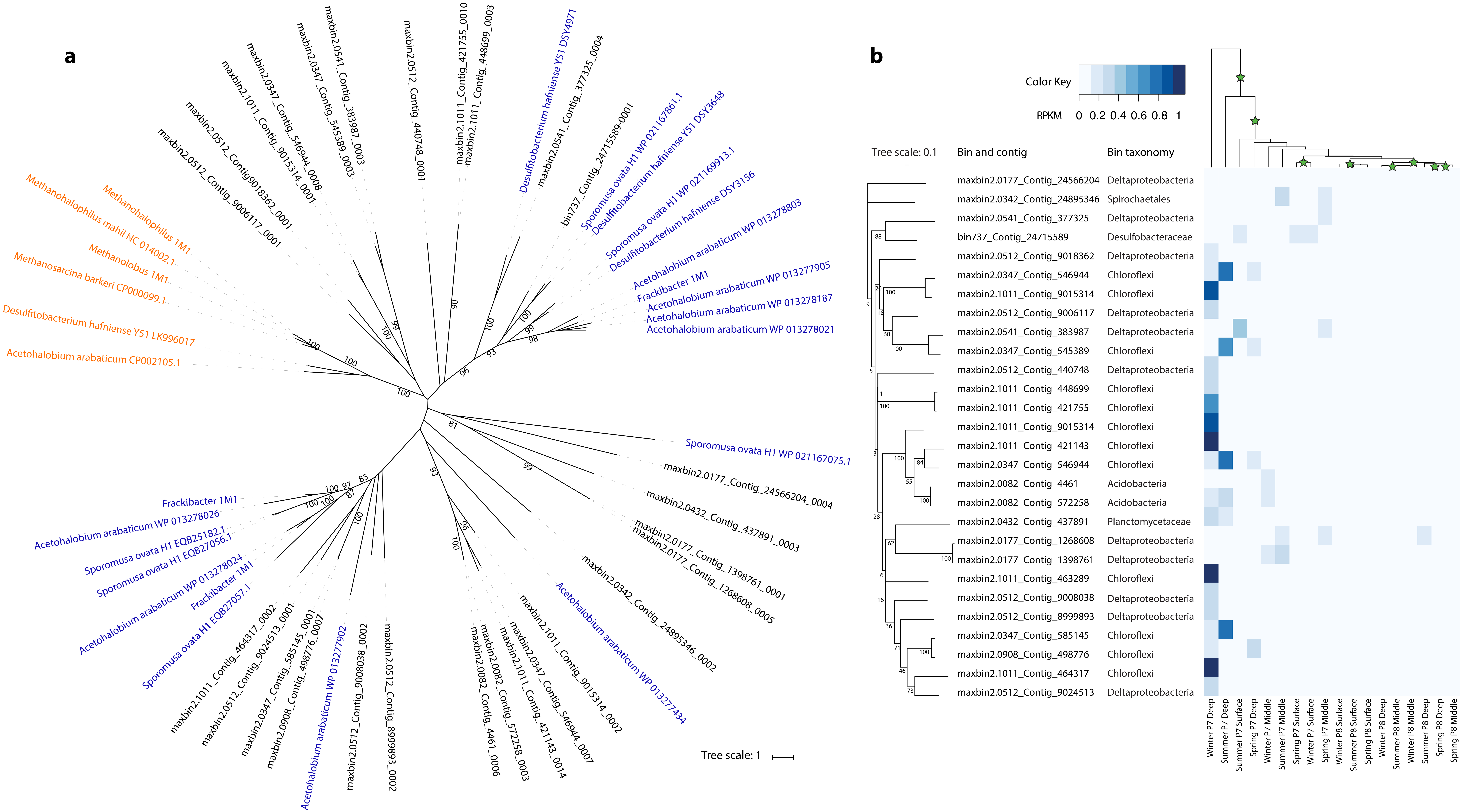

a
Methanohalophilus mahii NC 014002.1
Methanohalophilus 1M1
Methanolobus 1M1
Methanosarcina barkeri CP000099.1
Desulfitobacterium hafniense Y51 LK996017
Acetohalobium arabaticum CP002105.1
maxbin2.0512_Contig_9006117_0001
maxbin2.0512_Contig9018362_0001
maxbin2.1011_Contig_9015314_0001
maxbin2.0347_Contig_546944_0008
maxbin2.0347_Contig_545389_0003
maxbin2.0541_Contig_383987_0003
maxbin2.0512_Contig_440748_0001
maxbin2.1011_Contig_421755_0010
maxbin2.1011_Contig_448699_0003
Desulfitobacterium hafniense Y51 DSY4971
maxbin2.0541_Contig_377325_0004
bin737_Contig_24715589-0001
Sporomusa ovata H1 WP 021167861.1
Desulfitobacterium hafniense Y51 DSY3648
Sporomusa ovata H1 WP 021169913.1
Desulfitobacterium hafniense DSY3156
Acetohalobium arabaticum WP 013278803
Frackibacter 1M1
Acetohalobium arabaticum WP 013277905
Acetohalobium arabaticum WP 013278187
Acetohalobium arabaticum WP 013278021
Sporomusa ovata H1 WP 021167075.1
maxbin2.0177_Contig_24566204_0004
maxbin2.0432_Contig_437891_0003
maxbin2.0177_Contig_1398761_0001
maxbin2.0177_Contig_1268608_0005
maxbin2.0342_Contig_24895346_0002
Acetohalobium arabaticum WP 013277434
maxbin2.1011_Contig_9015314_0002
maxbin2.0347_Contig_546944_0007
maxbin2.1011_Contig_421143_0014
maxbin2.0082_Contig_572258_0003
maxbin2.0082_Contig_4461_0006
maxbin2.0512_Contig_8999893_0002
maxbin2.0512_Contig_9008038_0002
Acetohalobium arabaticum WP 013277902
maxbin2.0908_Contig_498776_0007
maxbin2.0347_Contig_585145_0001
maxbin2.0512_Contig_9024513_0001
maxbin2.1011_Contig_464317_0002
Sporomusa ovata H1 EQB27057.1
Frackibacter 1M1
Acetohalobium arabaticum WP 013278024
Sporomusa ovata H1 EQB27056.1
Sporomusa ovata H1 EQB25182.1
Acetohalobium arabaticum WP 013278026
Frackibacter 1M1
Tree scale: 1
b
Color Key
RPKM
0 0.2 0.4 0.6 0.8 1
Tree scale: 0.1
Bin and contig
Bin taxonomy
maxbin2.0177_Contig_24566204 Deltaproteobacteria
maxbin2.0342_Contig_24895346 Spirochaetales
maxbin2.0541_Contig_377325 Deltaproteobacteria
bin737_Contig_24715589 Desulfobacteraceae
maxbin2.0512_Contig_9018362 Deltaproteobacteria
maxbin2.0347_Contig_546944 Chloroflexi
maxbin2.1011_Contig_9015314 Chloroflexi
maxbin2.0512_Contig_9006117 Deltaproteobacteria
maxbin2.0541_Contig_383987 Deltaproteobacteria
maxbin2.0347_Contig_545389 Chloroflexi
maxbin2.0512_Contig_440748 Deltaproteobacteria
maxbin2.1011_Contig_448699 Chloroflexi
maxbin2.1011_Contig_421755 Chloroflexi
maxbin2.1011_Contig_9015314 Chloroflexi
maxbin2.1011_Contig_421143 Chloroflexi
maxbin2.0347_Contig_546944 Chloroflexi
maxbin2.0082_Contig_4461 Acidobacteria
maxbin2.0082_Contig_572258 Acidobacteria
maxbin2.0432_Contig_437891 Planctomycetaceae
maxbin2.0177_Contig_1268608 Deltaproteobacteria
maxbin2.0177_Contig_1398761 Deltaproteobacteria
maxbin2.1011_Contig_463289 Chloroflexi
maxbin2.0512_Contig_9008038 Deltaproteobacteria
maxbin2.0512_Contig_8999893 Deltaproteobacteria
maxbin2.0347_Contig_585145 Chloroflexi
maxbin2.0908_Contig_498776 Chloroflexi
maxbin2.1011_Contig_464317 Chloroflexi
maxbin2.0512_Contig_9024513 Deltaproteobacteria
Winter P7 Deep
Summer P7 Deep
Summer P7 Surface
Spring P7 Deep
Winter P7 Middle
Summer P7 Middle
Spring P7 Surface
Winter P7 Surface
Spring P7 Middle
Winter P8 Surface
Summer P8 Surface
Spring P8 Surface
Winter P8 Deep
Summer P8 Middle
Winter P8 Middle
Summer P8 Deep
Spring P8 Deep
Spring P8 Middle
